# Development and Mining of a Volatile Organic Compound Database

**DOI:** 10.1155/2015/139254

**Published:** 2015-09-30

**Authors:** Azian Azamimi Abdullah, Md. Altaf-Ul-Amin, Naoaki Ono, Tetsuo Sato, Tadao Sugiura, Aki Hirai Morita, Tetsuo Katsuragi, Ai Muto, Takaaki Nishioka, Shigehiko Kanaya

**Affiliations:** ^1^Graduate School of Information Science, Nara Institute of Science and Technology, 8916-5 Takayama, Ikoma, Nara 630-0192, Japan; ^2^School of Mechatronic Engineering, Universiti Malaysia Perlis (UniMAP), Ulu Pauh, 02600 Arau, Perlis, Malaysia; ^3^Department of Computer Science and Engineering, Toyohashi University of Technology, 1-1 Hibarigaoka, Tempaku, Toyohashi, Aichi 441-8580, Japan; ^4^Graduate School of Biological Science, Nara Institute of Science and Technology, 8916-5 Takayama, Ikoma, Nara 630-0192, Japan

## Abstract

Volatile organic compounds (VOCs) are small molecules that exhibit high vapor pressure under ambient conditions and have low boiling points. Although VOCs contribute only a small proportion of the total metabolites produced by living organisms, they play an important role in chemical ecology specifically in the biological interactions between organisms and ecosystems. VOCs are also important in the health care field as they are presently used as a biomarker to detect various human diseases. Information on VOCs is scattered in the literature until now; however, there is still no available database describing VOCs and their biological activities. To attain this purpose, we have developed KNApSAcK Metabolite Ecology Database, which contains the information on the relationships between VOCs and their emitting organisms. The KNApSAcK Metabolite Ecology is also linked with the KNApSAcK Core and KNApSAcK Metabolite Activity Database to provide further information on the metabolites and their biological activities. The VOC database can be accessed online.

## 1. Introduction

Recently big data has become an important topic that has significant roles to play in versatile disciplines of scientific research. Big data biology is a data-intensive science which has emerged because of the rapidly increasing volume of molecular biological data in omics fields such as genomics, transcriptomics, proteomics, and metabolomics [1–3]. With the explosively growing data scale, the development of biological databases incorporating different species has become a very important theme in big data biology. To address this need, we have developed KNApSAcK Family Databases (DBs), which have been utilized in a number of studies in metabolomics. The KNApSAcK Family Database systems previously have been used to understand the medicinal usage of plants based on traditional and modern knowledge [4, 5]. To facilitate a comprehensive understanding of the interactions between the metabolites of organisms and the chemical-level contribution of metabolites to human health, a Metabolite Activity DB known as the KNApSAcK Metabolite Activity DB has been constructed [6] and a network-based approach has been proposed to analyze the relationships between 3D structure and biological activities of the metabolites [7].

Metabolomics is the scientific study of quantification of profiles and analysis of chemical processes involving metabolites in a comprehensive fashion. In general, metabolites can be divided into two groups: primary and secondary metabolites. Primary metabolites are directly involved in the normal growth, development, and reproduction. On the other hand, secondary metabolites are not directly involved in these processes but usually have important ecological function, such as inter- or intraspecies communication, antifungal and antimicrobial activities, and also defense against pests and pathogens. Large portions of these defense compounds are volatile organic compounds (VOCs) that are involved in different ways of defense: direct defense and indirect defense. VOCs constitute only a small proportion of the total number of secondary metabolites produced by living organisms; however because of their important roles in chemical ecology specifically in the biological interactions between organisms and ecosystems, revealing and analyzing the roles of these VOCs is essential for understanding the interdependence of organisms [8].

VOCs originate from major pathways of secondary metabolisms of many living organisms, including human, animals, microorganisms, and plants. In plant kingdom, VOCs are responsible for internal and external communication between plants and herbivores, pathogens, pollinators, and parasitoids such as defense and attractant [9]. Microbial volatiles are widely used as biomarkers to detect human diseases [10]. This is because bacteria have a recognizable metabolism that produces bacteria-specific VOCs, which might be used for noninvasive diagnostic purposes [11]. For example, a volatile organic compound called methyl nicotine produced by* Mycobacterium tuberculosis* bacteria can be used as a noninvasive and rapid diagnostic marker for detection of Tuberculosis (TB) diseases [12]. Human also produces VOCs. Hundreds of volatiles are emitted from the human body in breath, blood, skin, and urine. These compounds reflect the different metabolic conditions of an individual [13]. Therefore, differences between the volatile profiles of individual humans can be used as an indicator to evaluate and monitor “disease” or “health” status. A review of breath analysis in disease diagnosis using volatile profiles is presented by Lourenço and Turner [14]. Breath analysis can be used as a biomarker to identify patients related to breast cancer [15], colorectal cancer [16], pulmonary tuberculosis [17], and lung cancer [18].

Advancements in analytical methods such as gas chromatography-mass spectrometry (GCMS), proton transfer reaction mass spectrometry (PTR-MS), and selected ion flow tube mass spectrometry (SIFT-MS) have provided an opportunity to identify the volatile metabolites of living organisms in research laboratories. These analytical approaches generate a large amount of data and require specialized mathematical, statistical, and bioinformatics tools to analyze such data. Despite the advances in sampling and detection by these analytical methods, only few databases have been developed to handle these large and complex datasets. There are few volatile organic compound databases which can be accessed freely; however their applicability is often limited by several elements. Most of these databases only focus on volatiles which are emitted by certain living organisms and have limited applications. For example, the Superscent database [19] only provides structure information of flavors and scents, and the mVOC database [20] provides information of microbial volatiles only. Flavornet [21] features compounds identified in experiments employing gas chromatography olfactometry (GC-O) analysis, and Pherobase [22] is focused on insect pheromones and semiochemicals. The vocBinBase [23] is a mass spectral database for volatiles which can allow for tracking and identification of volatile compounds in complex mixtures. None of these databases provide information on biological activities of VOCs and species-species interaction based on volatiles. Information on volatiles emission from microorganisms, plants, and other organisms is scattered in the literature until now, but there is no public and up-to-date database that accumulated comprehensive information of volatiles and their biological activities.

In the present study, we have developed a VOC database of microorganisms, fungi, and plants as well as human being, which comprises the relation between emitting species, the volatiles, and their biological activities. We have deposited the VOC data into KNApSAcK Metabolite Ecology Database and this database is currently available at http://kanaya.naist.jp/MetaboliteEcology/top.jsp. Apart from the database development, we also analyzed the VOC data using hierarchical clustering and network clustering based on DPClus [24, 25]. In addition, we also performed the heatmap clustering based on Tanimoto coefficient as the similarity index of the chemical structure to cluster all VOCs emitted by various biological species to understand the relationships between chemical structures of VOCs and their biological activities.

## 2. Methods

### 2.1. Data Collection and Database Development

The data were collected by an extensive literature search up to January 2015 on PubMed (http://www.ncbi.nlm.nih.gov/pubmed) and Google Scholar (http://scholar.google.co.jp/). The PubMed search provided 60 articles based on the keywords “volatile organic compounds” and “metabolites.” The information on VOCs, emitting species, target species, and their biological activities was extracted and deposited into KNApSAcK Metabolite Ecology Database. The KNApSAcK Metabolite Ecology is also linked to the KNApSAcK Core and KNApSAcK Metabolite Activity Database to provide further information on the metabolites and their biological activities. Data were divided into two types: (1) microorganisms species-VOC binary relations; (2) emitting species-VOC-target species triplet relations.

### 2.2. Clustering of Species Based on VOC Similarity

Clustering is an unsupervised learning method, which is the task of grouping a set of objects into the same group (cluster) based on similarity or distance measures. This technique is important for knowledge discovery and has been applied in many applications such as machine learning, pattern recognition, image analysis, and bioinformatics [26–28]. In this study, we utilized hierarchical clustering and graph clustering methods for classifying the VOC emitting species. Both methods are discussed separately in the following.

#### 2.2.1. Hierarchical Clustering

We used hierarchical agglomerative clustering method, which starts out by putting each observation into its own separate cluster. It then examines all the distances between all the observations and pairs together the two closest ones to form a new cluster. The process continues until all the observations are included in a single cluster. The result of clustering is usually represented by a dendrogram. In our case, we used a species versus VOC matrix. Let this matrix be called *M* and *M*_*ik*_ = 1 if the species *i* is related to the *k*th VOC or otherwise *M*_*ik*_ = 0. Hierarchical methods require a distance matrix and hence we determined the Euclidean distances between species. Euclidean distance *d* between species *i* and species *j* can be calculated as follows:

(1) $$\begin{array}{c} d\left(\;i,j\;\right)=\sqrt{\overset{n}{\underset{k=1}{\sum }}{\left(M_{ik}-M_{jk}\right)}^{2}}. \end{array}$$

Here, *n* is the number of VOCs and there are 1088 VOCs in our data. Based on Euclidean distance, we perform Ward's hierarchical clustering analysis using *R*, an open source programming language.

#### 2.2.2. Graph Clustering Based on DPClus

DPClus is a graph clustering software [24], which has been developed based on a graph clustering algorithm that can extract densely connected nodes as a cluster [25]. This algorithm can be applied to an undirected simple graph *G* = (*N*, *E*) that consists of a finite set of nodes *N* and a finite set of edges *E*. Two important parameters are used in this algorithm, which are density *d*_*k*_ and cluster property *cp*_*nk*_. Density *d*_*k*_ of any cluster *k* is the ratio of the number of edges present in the cluster (|*E*|) and the maximum possible number of edges in the cluster (|*E*|_max_). The cluster property of node *n* with respect to cluster *k* is represented by

(2) $$\begin{array}{c} {cp}_{nk}=\frac{E_{nk}}{d_{k}\times N_{k}} \end{array}$$

*N*_*k*_ is the number of nodes in cluster *k*. *E*_*nk*_ is the total number of edges between the node *n* and each of the nodes of cluster *k*. In this study, we apply the DPClus algorithm to identify certain groups of microorganism species based on VOC similarity. A network is constructed where a node represents a microorganism species and an edge indicates high VOC similarity between the corresponding species pair. We selected 5% of the organism pairs based on lower Euclidean distance between them. We used the nonoverlapping mode with the following DPClus settings: Cluster property *cp*_*nk*_ was set to 0.5, density value *d*_*k*_ was set to 0.6, and minimum cluster size was set to 2.

### 2.3. Clustering of VOCs Based on Chemical Structure Similarity

We also performed a classification of VOCs based on their chemical structure similarity. In order to determine the similarity between two chemical compounds, we used Tanimoto coefficient as similarity measure. The Tanimoto coefficient is defined as (3), which is the proportion of the features shared between two compounds divided by their union [29]

(3) $$\begin{array}{c} {Tanimoto}_{A,B}=\frac{AB}{A+B-AB}. \end{array}$$

The variable *AB* is the number of features (or on-bits in binary fingerprint) common in both compounds, while *A* and *B* are the number of features that are related to individual compounds, respectively. The Tanimoto coefficient has a range from 0 to 1 with higher values indicating greater similarity than lower ones. Additionally, a Tanimoto coefficient value larger than 0.85 indicates that the compared compounds may have similar biological activity [30]. For the purpose of calculating Tanimoto coefficient, it is obligatory to assign fingerprints to the compounds. ChemMine package in R was used to generate atom pair fingerprints and calculation of Tanimoto coefficient [31, 32]. 2D compound structures in the generic structure definition file (SDF) format were obtained from PubChem database (https://pubchem.ncbi.nlm.nih.gov) and then were imported into ChemmineR package in one batch file. The atom pair descriptors are calculated during the SDF import and stored in a searchable descriptor database as a list object.

Based on Tanimoto similarity measure between chemical structures, heatmap clustering was performed for classifying the VOCs. We also determined the *p* values of the clusters based on hypergeometric distribution using

(4) $$\begin{array}{c} p\;\;value=1-\overset{K-1}{\underset{i=0}{\sum }}\frac{\left(\begin{array}{c} V\\ i \end{array}\right)\left(\begin{array}{c} N-V\\ C-i \end{array}\right)}{\left(\begin{array}{c} N\\ C \end{array}\right)}. \end{array}$$

Here *N* is the total number of VOCs, *C* is the size of a cluster, and *V* and *K*, respectively, are the number of VOCs of a certain category in the whole data and in the cluster. The hypergeometric distribution is used to calculate the statistical significance of having drawn specific *K* successes (out of *N* total draws) from the whole population. The test is often used to identify which subpopulations are over- or underrepresented in a sample. The calculated *p* value implies the probability of getting *K* or more VOCs of a particular category in a cluster when the cluster is formed by random selection. Lower *p* value indicates that the statistical significance is high.

Our purpose is to relate a structure group to a biological activity if and only if the structure group is overrepresented by VOCs associated with that biological activity.

## 3. Results and Discussion

### 3.1. KNApSAcK VOC Database

**Figure 1:**
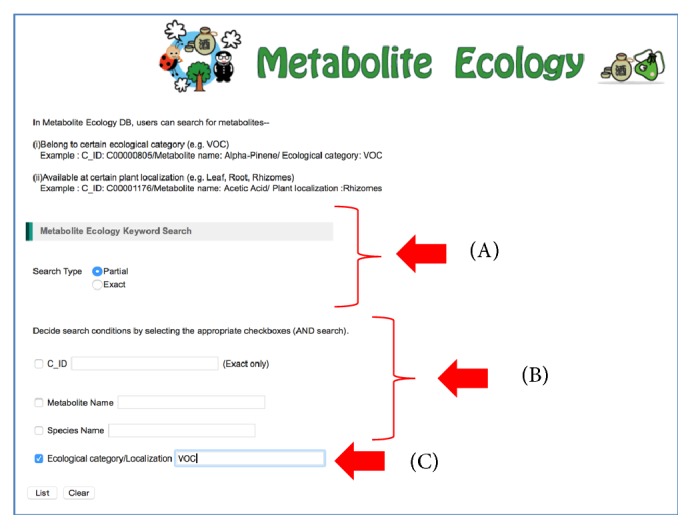
The main window of the KNApSAcK Metabolite Ecology Database. (A) Section used to select the search type. (B) Section used to select the search conditions and to input keywords. (C) Users can input “VOC” in the text box for the ecological/localization category to search VOC data.

**Figure 2:**
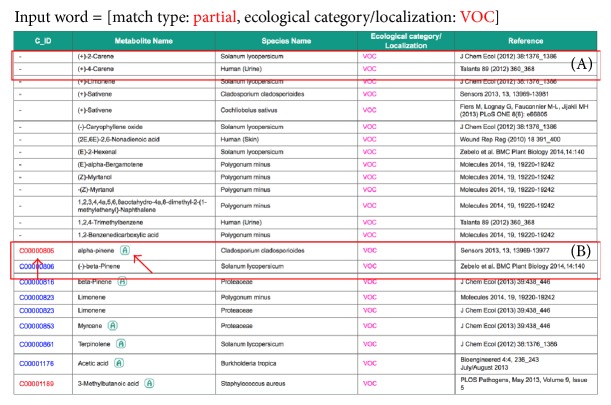
The results retrieved for VOCs search in the KNApSAcK Metabolite Ecology Database. (A) The VOC, which does not have a KNApSAcK compound ID. (B) The VOC, which has a KNApSAcK compound ID. Users can click at the C_ID to find more information on the metabolite and at the button “A” to retrieve its biological activity.

**Figure 3:**
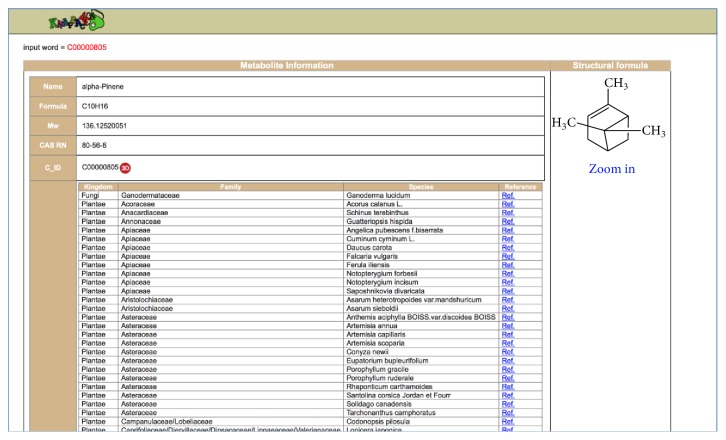
An example of the search results obtained by clicking the C_ID, C00000805, which were retrieved from the KNApSAcK Core Database.

**Figure 4:**
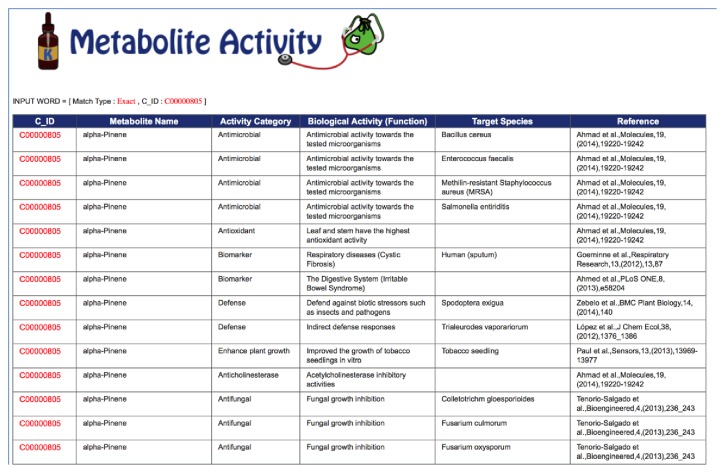
An example of the search result of biological activity related to C_ID C00000805, which were retrieved from the KNApSAcK Metabolite Activity Database.

At present, we have accumulated 1088 VOCs emitted by 517 microorganisms species and 341 VOCs emitted by other biological species including plants, animals, and human. These VOC data have been deposited into KNApSAcK Metabolite Ecology Database, which allows users to search information on VOCs using the KNApSAcK compound ID and metabolite name. This KNApSAcK Metabolite Ecology Database is also linked to the KNApSAcK Core and KNApSAcK Metabolite Activity Database to provide further information on the volatile metabolites and their biological activities. The VOC database can be accessed online at http://kanaya.naist.jp/MetaboliteEcology/top.jsp.  Figure 1 shows the main window of the KNApSAcK Metabolite Ecology Database, which shows the search type and search condition. For search type, users can choose either partial or exact string matching searches by clicking the corresponding button, that is, partial or exact (Figure 1(A)). Other check boxes can also be selected to specify different search conditions (Figure 1(B)) such as KNApSAcK compound ID (C_ID), metabolite name, species name, and ecological category or localization. To search VOC data, users can input “VOC” in the text box for the ecological category/localization category, select the corresponding check box, and then click the List button (Figure 1(C)). Part of the results retrieved by entering “VOC” in the text box is shown in Figure 2. The attributes in the list are C_ID, which corresponds to the KNApSAcK compound ID, metabolite name, species name (VOCs emitting species), ecological category/localization (VOC), and references (the source of the VOCs information), from left to right. During the literature search, it turned out that many VOCs do not have a KNApSAcK compound ID but might be biologically relevant. Therefore, those VOCs were also included into the database. For example, in the first line, the VOC with the name (+)-2-Carene (Figure 2(A)) does not have a KNApSAcK compound ID; however it was produced by* Solanum lycopersicum.* In the future, we will find more information on these VOCs and assign the KNApSAcK compound ID to these metabolites. On the other hand, information related to the VOCs that have KNApSAcK compound ID can be obtained by clicking the C_ID as in Figure 2(B).  Figure 3 shows the search results obtained by clicking the C_ID, C00000805, which were retrieved from the KNApSAcK Core Database. Users can retrieve further knowledge of this metabolite, such as molecular formula, molecular weight, CAS RN, 3D structure, and other species information, which also produce the corresponding metabolite. To understand the relationships between VOCs and their biological activities, we also integrate the KNApSAcK Metabolite Ecology Database with KNApSAcK Metabolite Activity Database. Information on VOCs related to biological activity can be obtained by clicking the “A” button as in Figure 2(B).  Figure 4 shows the search result of biological activity related to C_ID C00000805, which were retrieved from the KNApSAcK Metabolite Activity Database. The attributes in the list are C_ID, metabolite name, activity category, biological activity, target species, and references, from left to right. Here, the metabolite known as* alpha-pinene *(C_ID C00000805) has few biological activity categories such as antimicrobial, antioxidant, biomarker, defense, plant growth enhancement, anticholinesterase, antifungal, dermatitic, irritant, psychotomimetic, and toxic.

### 3.2. Clustering of Microorganisms Based on VOC Similarity

**Figure 5:**
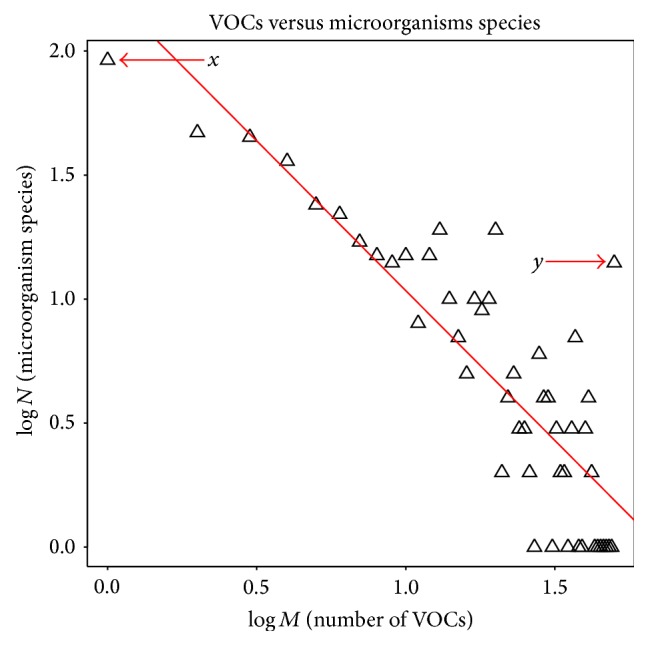
The log-log relation between the number of VOCs and the number of related microorganisms' species.

**Figure 6:**
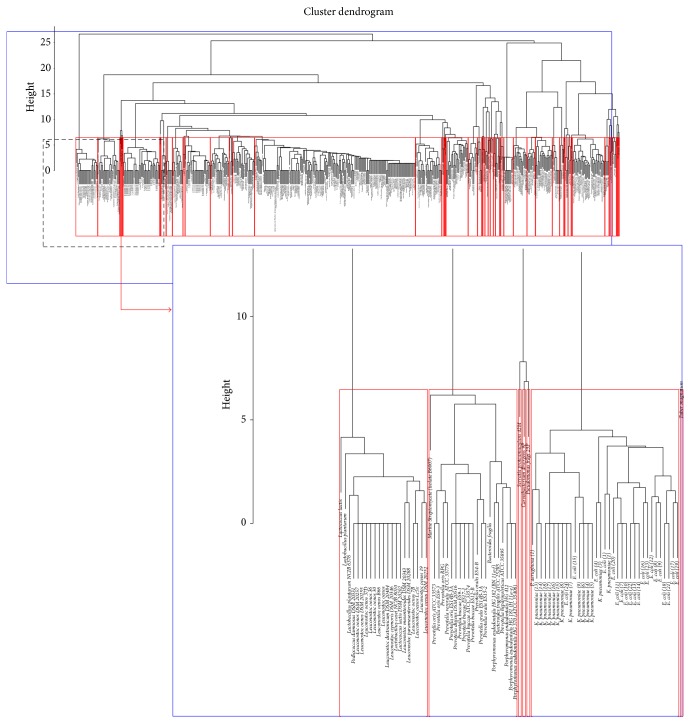
Hierarchical dendrogram plot of microorganism species based on VOC presence.

Initially, the accumulated VOC data were divided into two types: (1) microorganisms species-VOC binary relations; (2) emitting species-VOC-target species triplet relations. This section focuses on the clustering analysis result of the first type of data, which is the relationship between microorganism species and their emitting VOCs. Until now, we have accumulated 1088 compounds produced by 517 microorganisms.  Figure 5 shows the log-log relation between the number of VOCs, *M*, and the frequency of species, *N*. The pattern roughly follows power law [33].  Figure 5 shows that there are 92 species that emit only one type of VOC (Point *x*). Highest 50 types of VOCs are emitted by an individual species and there are 14 such species in our present data (Point *y*). From this statistical analysis, we can say that most microorganism species emit a few VOCs, which can act as their odor fingerprint. The information of emitting species and compounds has been converted into a 517 × 1088 binary matrix (“1” indicates presence while “0” indicates absence). The binary matrix then was used to calculate the Euclidean distance between species. From the Euclidean distance, hierarchical clustering of species was performed.  Figure 6 shows a hierarchical dendrogram plot of microorganism species based on VOC presence. Here, we cut the dendrogram tree to 50 clusters and the threshold height for this clustering is 7. Supplementary Table  1 (in Supplementary Material available online at http://dx.doi.org/10.1155/2015/139254) shows the species name with their corresponding clusters and the pathogenicity of the microorganism species. Interestingly, 77 species from 517 species are known as pathogenic bacteria and are classified into six clusters, which are clusters 6, 27, 35, 40, 47, and 48. Out of these six clusters, three clusters, that is, clusters 35, 40, and 47, contain 100% pathogenic bacterial species such as* P. aeruginosa*,* K. pneumoniae,* and* E. coli*. The other three clusters contain both pathogenic and nonpathogenic species. For example, cluster 6 consists of 11 (7.2%) pathogenic bacterial species while cluster 27 comprises only one (7.7%) pathogen species. Cluster 48 contains 4 (16%) pathogenic bacterial species. Out of all 50 clusters, the rest of 44 clusters contain nonpathogenic species. These results imply that VOCs emitted by some pathogenic bacteria are different from those emitted by nonpathogenic bacteria. These results show consistency between VOC and pathogenicity based classification of microorganisms.

**Figure 7:**
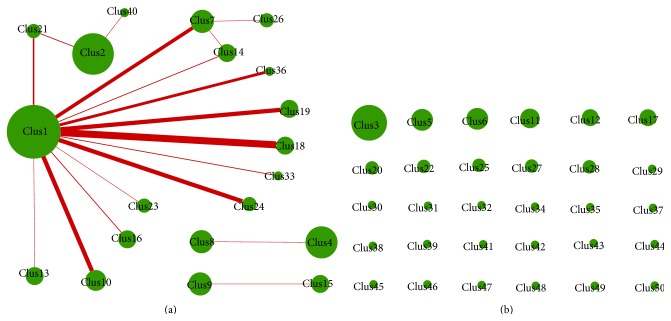
Hierarchical graph of DPClus clustering result. (a) Connected nodes. (b) Independent nodes.

In order to extract different and more information, we constructed a network by inserting edges between species for which the Euclidean distance is less than a threshold. The threshold was decided to include the lowest 5% distances as edges in the network. We then determined the high-density clusters in that network by applying the graph clustering algorithm DPClus. Supplementary Figure  1 shows the overall network, which displays all the generated clusters in such a way that intracluster edges are green and intercluster edges are red.  Figure 7(a) shows the hierarchical connected graph of the clustering result, where the green nodes represent clusters of microorganism species and the red edges represent the interaction between clusters. The radius of a green node in the hierarchical graph in Figure 7 is proportional to the logarithm of the number of nodes in the cluster it represents. The width of a red edge in the hierarchical graph between a pair of clusters is proportional to the number of edges between those clusters in the original graph.  Figure 7(b) shows the independent nodes of the hierarchical graph, which indicates that these clusters do not interact with other clusters.

Overall, DPClus generated 50 clusters where 20 clusters are connected nodes to each other while the remaining 30 clusters are independent nodes. Only cluster 1 contains both pathogenic and nonpathogenic microorganisms. Clusters 2, 7, 14, 21, 26, and 40 consist of only pathogenic bacteria while the other clusters are consisting of only nonpathogenic bacteria. These results imply that pathogenicity of microorganisms can be linked to characteristic combinations of identical VOCs emitted by them. Some of the pathogenic members of cluster 1 such as* Klebsiella pneumoniae*,* Escherichia coli*,* Staphylococcus aureus,* and* Pseudomonas aeruginosa* are very highly connected to other pathogenic clusters, for example, clusters 2 and 7.  Figure 7(a) shows that clusters 2, 7, 14, 21, 26, and 40 are connected by red edges, which reflect VOC similarity between pathogenic microorganisms. Also, there is VOC based similarity between nonpathogenic species of cluster 1 and clusters 10, 13, 16, 18, 19, 23, 24, 33, and 36. The red edges between clusters 4 and 8 and between clusters 9 and 15 are also because of VOC similarity between nonpathogenic species of those clusters. Here it is noteworthy that the rest of 30 clusters consisting of nonpathogenic species are independent clusters, which implies that many nonpathogenic groups of species emit quite unique types of VOCs as shown in Figure 7(b). Supplementary Figure  2 shows the microorganism species belong to cluster 1 (pathogenic and nonpathogenic), cluster 7 (pathogenic only), and cluster 10 (nonpathogenic species only), respectively. Here the internal nodes of a cluster are shown connected by green edges and its neighboring clusters are shown connected by red edges. To evaluate the stability of graph clustering results by DPClus, we also clustered the networks generated by several random samplings of 80% or more edges of the original network. We found that DPClus can still cluster the microorganisms species based on pathogenicity.

The results of network clustering and hierarchical clustering are similar in the sense that both results indicated that VOC based classification of microorganisms is consistent with their classification based on pathogenicity. However, clustering by DPClus further revealed existence and nonexistence of relations between different pathogenic and nonpathogenic groups of microorganisms.

### 3.3. Clustering Analysis of VOCs Based on Chemical Structure Similarity

**Figure 8:**
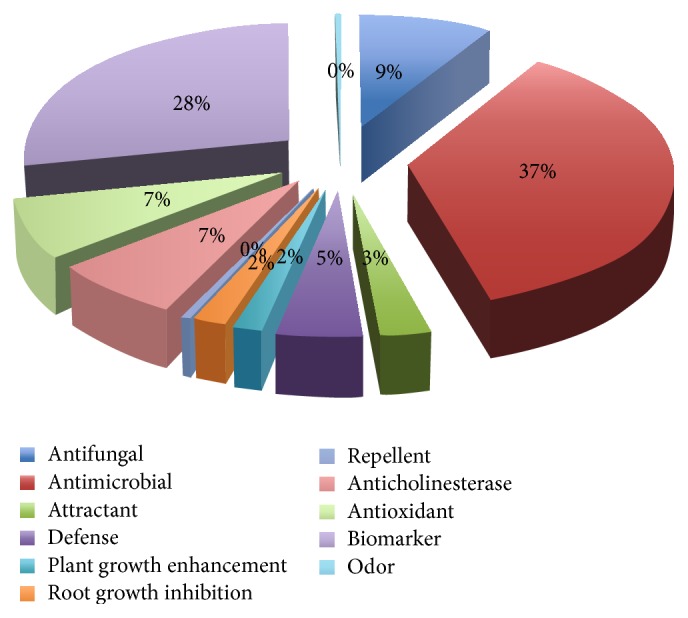
Pie chart showing the relative frequencies VOCs belonging to 11 biological activities.

**Figure 9:**
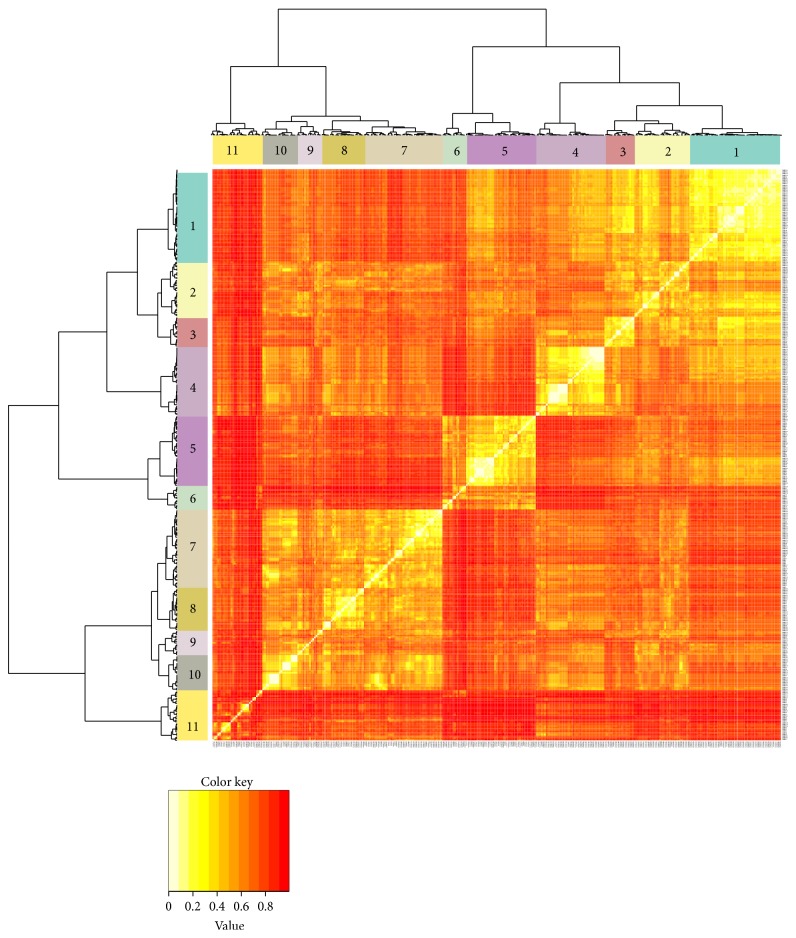
Heatmap clustering of VOCs based on chemical structure similarity determined by Tanimoto coefficient.

The first type of data focused on microorganism species only but the second type of data includes VOCs emitted by other biological species such as plants, animals, and humans. The second data type that we have accumulated until now is 1044 species-species interactions via 341 VOCs associated with 11 groups of biological activities. The biological activities of VOCs are classified into two types: (i) chemical ecology related activities, in which most VOCs are involved in interaction between species for survival of organisms such as defense and antimicrobial, and (ii) human health care related activities, in which many VOCs are widely used as disease biomarker and odor. From our accumulated data, 57.3% of the activities belong to chemical ecology such as antifungal, antimicrobial, attractant, defense, plant growth enhancement, root growth inhibition, and repellent activities and 42.7% are human health related activities such as disease biomarker, odor, anticholinesterase, and antioxidant as shown in Figure 8. There are many VOCs, which have several biological activities. Thus, it is important to investigate the relationships between VOCs and their biological activities statistically. Initially, we determined pairwise chemical structural similarity between VOCs based on Tanimoto coefficient. 2D compound structures in the generic structure definition file (SDF) format of all 341 VOCs were obtained from PubChem database (https://pubchem.ncbi.nlm.nih.gov) and then were imported into ChemmineR package in one batch file. We calculated the chemical structure similarity using Tanimoto coefficient. Then, we converted the Tanimoto similarity matrix into distance matrix by subtracting each of the similarity values from 1. Based on distance matrix, we performed heatmap clustering and the result is shown in Figure 9. White and red colours indicate the extreme distance values of 0 and 1, respectively, and the intermediate distance values are indicated by the intensity of the red colour.

From the heatmap plot, we tentatively outlined 11 clusters of VOCs. The count of VOCs belonging to each activity group in each cluster is shown in Table 1. To assess the richness of VOCs of similar activity in individual clusters, we determined their *p* values based on hypergeometric distribution which are also shown in Table 1. The major types of chemical compounds belonging to each cluster and their corresponding biological activities are mentioned in Table 2. The chemical structures of the VOCs belonging to all clusters (cluster 1 to cluster 11) are shown in Supplementary Figure  2.

From this result, we can see that there are 55 VOCs belonging to cluster 1 and mainly involved with anticholinesterase, antimicrobial, antioxidant, and defense activities, for example, beta-caryophyllene, isocaryophyllene, and caryophyllene. All compounds in cluster 1 are terpenoids, of which 15 VOCs are monoterpenoids (10 carbon units) and 40 VOCs are sesquiterpenoids (15 carbon units). There are 33 VOCs in cluster 2 and the *p* values corresponding to anticholinesterase, antimicrobial, and antioxidant are 4.849 × 10^−4^, 9.696 × 10^−4^, and 4.849 × 10^−4^, respectively. Some of the VOCs that are classified into cluster 2 are monoterpenoids and sesquiterpenoids such as beta-linalool, terpinen-4-ol, p-menth-1-en-8-ol, drimenol, and nerolidol. 17 VOCs are alcohol, aldehyde, ketone, epoxide, and ester of terpenoids. The other VOCs are alcohol, aldehyde, carboxylic acid, ester, and ketone of straight-chain alkenes.

For cluster 3, there are 41 compounds and the main biological activity involved is biomarker for various diseases such as colorectal cancer and asthma. We obtained small *p* value (1.835 × 10^−3^) for biomarker activity of cluster 3. All compounds are alkanes; most of them are emitted in human breath such as octane, isobutane, 2-methylpentane, methylcyclohexane, hexane, and cyclohexane.

There are 18 compounds in cluster 4 and all of them are alkenes such as beta-farnesene, alpha-caryophyllene, ocimene, and beta-ocimene. These compounds are mainly associated with chemical ecology activity, which is antifungal and the *p* value for this activity is 2.561 × 10^−2^. For cluster 5, there are 21 VOCs which are aldehyde, ester, carboxylic acid, and ketone of C8–C18 alkanes. Cluster 5 is significantly related with multiple biological activities, which are anticholinesterase, antimicrobial, antioxidant, biomarker, and repellent activities.

There are 25 VOCs in clusters 6 and 21, of them are alcohol and ether of C3–C8 alkanes. We also obtained small *p* value for plant growth enhancement activity (3.531 × 10^−3^), root growth inhibition activity (4.111 × 10^−2^), and odor activity (2.29 × 10^−5^) for cluster 6. An example of VOCs involved in plant growth enhancement activity is 2,3-butanediol and there are many reports that this compound released by soil microorganisms had improved plant growth and increased pathogen resistance [34, 35]. For odor activity, compounds involved are in alcohol sulfanylalkanols chemical class group such as 2-methyl-3-sulfanylbutan-1-ol and 3-methyl-3-sulfanylhexan-1-ol. These compounds have a pungent sweat/kitchen odor, also reminiscent of onions with some fruity connotations which are transformed into the volatile substances by bacterial enzymes present only in corynebacteria.

There are 47 VOCs in clusters 7 and 45, of them are ester, carboxylic acid, ketone, and aldehyde of noncyclic C2–C9 alkanes. Cluster 7 is significantly related with multiple biological activities, which are attractant (*p* value = 3.829 × 10^−2^) and biomarker for various diseases (*p* value = 4.42 × 10^−5^). Aldehydes belong to cluster 7 such that acetaldehyde, propanal, hexanal, 2-methyl-butanal, pentanal, heptanal, and 3-methyl-butanal are mostly used as biomarker for various diseases including cancers and irritable bowel syndrome. In cluster 8, there are 15 VOCs belonging to this cluster, which consist of epoxide, ethers, esters, and alcohols. In cluster 9, there are 42 VOCs and the main biological activity is attractant (*p* value = 1.983 × 10^−2^). All VOCs belonging to cluster 9 are aromatic compounds, in which 24 VOCs are aromatic alcohols, carboxylic acids, esters, ketones, and ethers. 16 VOCs are aromatic compounds consisting of C and H atoms. One VOC consists of C, H, and Br atoms. One VOC is an alkane ester. Also, there are 14 VOCs in cluster 10 which are aromatic compounds. 12 VOCs are heteroaromatic compounds that consist of one or more sulfur, nitrogen, or oxygen atoms. On the other hand, 30 VOCs belonging to cluster 11 are of diverse types of C0–C6 small molecules with low molecular weight, ranging from hydrogen cyanide (27.02534) to tetrachloroethyene (165.8334). The main biological activity for cluster 10 and cluster 11 is biomarker for various diseases. The *p* values for biomarker activity for cluster 10 and cluster 11 are 1.036 × 10^−2^ and 1.963 × 10^−3^, respectively. The major VOCs involved in this activity are isoxazole, 2,3-dimethyl-pyrazine, and 2-methyl-pyrazine which are mostly produced in human urine and can be used as biomarker for autism spectrum disorders.

The heatmap clustering shows that there are strong links between chemical structure of VOCs and their biological activities. Comparative activity relationships between chemical ecology and human health care activity will lead to systematization of metabolomics combined with human and ecological metabolic pathways.

## 4. Conclusion

In the present study, we have developed a database of VOCs emitted by various living organisms including microorganisms, plants, animals, and human, which can be accessed at KNApSAcK Metabolite Ecology Database. Apart from VOC biological activities related to human health care, more than half of the biological activities are associated with chemical ecology. Hierarchical clustering and graph clustering by DPClus algorithm were utilized to extract specific microorganism species clusters based on VOC similarity. We found consistency between VOC and pathogenicity based classification of microorganisms. Additionally, heatmap clustering based on Tanimoto similarity measure was used to cluster the VOCs emitted by various species. We found that similar chemical structures of VOCs indicate possibilities of exhibiting similar biological activities. In future work, we will accumulate more data and perform comprehensive analysis of the VOCs in the context of human health care and chemical ecology. The KNApSAcK Metabolite Ecology Database may be useful for the discovery of novel agriculture tools and also for the noninvasive identification of biomarkers in medical diagnostic field as well as systematic research in various omics fields, especially metabolomics integrated with ecosystems.

## Supplementary Material

Supplementary Material includes a Supplementary Table and 3 Supplementary Figures. The Supplementary Table contains a list of species name with their corresponding clusters and the pathogenicity of the microorganism species. The Supplementary Figures contain overall network generated by DPClus algorithm (Supplementary Figure 1), the three example clusters of microorganism species that classify the microorganism species according to their pathogenicity (Supplementary Figure 2) and chemical structures of VOCs belonging to all clusters (Supplementary Figure 3).

## Figures and Tables

**Table 1:** The count of VOCs belonging to each activity group in each cluster and their *p* value based on hypergeometric distribution.

Biological activity	Cluster ID (Count)	Cluster 1 (55)	Cluster 2 (33)	Cluster 3 (41)	Cluster 4 (18)	Cluster 5 (21)	Cluster 6 (25)	Cluster 7 (47)	Cluster 8 (15)	Cluster 9 (42)	Cluster 10 (14)	Cluster 11 (30)
Anticholinesterase	*p* value (count)	5.28 × 10^−7^ (26)	4.849 × 10^−4^ (15)	0.6181 (8)	0.9109 (2)	1.274 × 10^−2^ (9)	0.9147 (3)	1 (0)	0.3596 (4)	0.9994 (2)	1 (0)	1 (0)

Antifungal	*p* value (count)	0.9128 (2)	0.9115 (1)	0.5399 (3)	2.561 × 10^−2^ (4)	1 (0)	0.5176 (2)	0.6403 (3)	0.6571 (1)	0.3099 (4)	1 (0)	0.3291 (3)

Antimicrobial	*p* value (count)	2.10 × 10^−6^ (26)	9.696 × 10^−4^ (15)	0.6898 (8)	0.9281 (2)	1.871 × 10^−2^ (9)	0.8246 (4)	0.9999 (1)	0.4049 (4)	0.9997 (2)	1 (0)	1 (0)

Antioxidant	*p* value (count)	5.28 × 10^−7^ (26)	4.849 × 10^−4^ (15)	0.6181 (8)	0.9109 (2)	1.274 × 10^−2^ (9)	0.9147 (3)	1 (0)	0.3596 (4)	0.9994 (2)	1 (0)	1 (0)

Attractant	*p* value (count)	0.9708 (2)	0.8144 (2)	1 (0)	0.4831 (2)	1 (0)	0.1661 (4)	3.829 × 10^−2^ (8)	0.1356 (3)	1.983 × 10^−2^ (8)	1 (0)	1 (0)

Biomarker	*p* value (count)	1 (8)	0.9999 (11)	1.835 × 10^−3^ (34)	0.7944 (10)	1.444 × 10^−2^ (18)	0.9821 (11)	4.42 × 10^−5^ (41)	0.9948 (5)	6.071 × 10^−2^ (31)	1.036 × 10^−2^ (13)	1.963 × 10^−3^ (26)

Defense	*p* value (count)	3.35 × 10^−9^ (22)	9.258 × 10^−2^ (7)	0.9758 (2)	0.6764 (2)	0.7594 (2)	0.8418 (2)	0.9987 (1)	0.8668 (1)	0.9787 (2)	1 (0)	1 (0)

Plant growth enhancement	*p* value (count)	6.01 × 10^−2^ (4)	1 (0)	1 (0)	1 (0)	1 (0)	3.531 × 10^−3^ (4)	0.7778 (1)	1 (0)	1 (0)	1 (0)	0.6069 (1)

Root growth inhibition	*p* value (count)	0.1749 (5)	0.8632 (1)	0.9183 (1)	1 (0)	0.7111 (1)	4.111 × 10^−2^ (4)	0.7672 (2)	0.5847 (1)	0.7062 (2)	0.5591 (1)	0.8347 (1)

Odor	*p* value (count)	1 (0)	1 (0)	1 (0)	1 (0)	1 (0)	2.29 × 10^−5^ (4)	1 (0)	1 (0)	1 (0)	1 (0)	1 (0)

Repellent	*p* value (count)	1 (0)	7.551 × 10^−2^ (2)	1 (0)	1 (0)	1.871 × 10^−3^ (3)	1 (0)	1 (0)	1 (0)	1 (0)	1 (0)	1 (0)

**Table 2:** Summary of clustering result and its descriptions related to chemical structures and biological activities.

Cluster ID (count)	Description on chemical structures	Related biological activities
Cluster 1 (55 VOCs)	All compounds are terpenoids. 15 VOCs are monoterpenoids (10 carbon units) and 40 VOCs are sesquiterpenoids (15 carbon units).	Anticholinesterase, antimicrobial, antioxidant, and defense.

Cluster 2 (33 VOCs)	17 VOCs are alcohol, aldehyde, ketone, epoxide, and ester of terpenoids. The other VOCs are alcohol, aldehyde, carboxylic acid, ester, and ketone of straight-chain alkenes.	Anticholinesterase, antimicrobial, and antioxidant.

Cluster 3 (41 VOCs)	Alkanes.	Biomarker.

Cluster 4 (18 VOCs)	Alkenes.	Antifungal.

Cluster 5 (21 VOCs)	Aldehyde, ester, carboxylic acid, and ketone of C8–C18 alkanes.	Anticholinesterase, antimicrobial, antioxidant, biomarker, and repellent.

Cluster 6 (25 VOCs)	21 VOCs are alcohol and ether of C3–C8 alkanes.	Plant growth enhancement and root growth inhibition and odor.

Cluster 7 (47 VOCs)	45 VOCs are ester, carboxylic acid, ketone, and aldehyde of noncyclic C2–C9 alkanes.	Attractant and biomarker.

Cluster 8 (15 VOCs)	VOCs consist of epoxide, ethers, esters, and alcohols.	—

Cluster 9 (42 VOCs)	24 VOCs are aromatic alcohols, carboxylic acids, esters, ketones, and ethers. 16 VOCs are aromatic compounds consisting of C and H atoms. One VOC consists of C, H, and Br atoms. One VOC is an alkane ester.	Attractant.

Cluster 10 (14 VOCs)	Aromatic compounds. 12 VOCs are heteroaromatic compounds that consist of one or more sulfur, nitrogen, or oxygen atoms.	Biomarker.

Cluster 11 (30 VOCs)	VOCs are quite diverse in chemical elements, C0–C6 small molecules.	Biomarker.
