# Supplementary material for: Development and Mining of a Volatile Organic Compound Database
Source: Biomed Res Int. 2015 Sep 30;2015:139254. doi: 10.1155/2015/139254 (PMC4606137; doi:10.1155/2015/139254)
Supplement: Supplementary file 1 — Supplementary Material includes a Supplementary Table and 3 Supplementary Figures. The Supplementary Table contains a list of species name with their corresponding clusters and the pathogenicity of the microorganism species. The Supplementary Figures contain overall network generated by DPClus algorithm (Supplementary Figure 1), the three example clusters of microorganism species that classify the microorganism species according to their pathogenicity (Supplementary Figure 2) and chemical structures of VOCs belonging to all clusters (Supplementary Figure 3). [file 139254.f1.pdf]

Supplementary Table 1 Microorganisms species name corresponding to the clusters and their pathogenicity.

Species No	Species Name	Cluster No	Pathogenicity
1	Chondromyces crocatus	1	Non-pathogenic
2	Nannocystis exedens	2	Non-pathogenic
3	Nannocystis exedens Na eB37	2	Non-pathogenic
4	Nannocystis exedens subsp. cinnabarina Na c29	2	Non-pathogenic
5	Tuber magnatum	3	Non-pathogenic
6	Phoma sp.	4	Non-pathogenic
7	Tuber melanosporum	5	Non-pathogenic
8	Penicillium roqueforti (IBT 16404)	6	Non-pathogenic
9	Nannocystis exedens Na e485	6	Non-pathogenic
10	Streptomyces citreus	6	Non-pathogenic
11	Acremonium furcatum BAFC 51375	6	Non-pathogenic
12	Bacillus strains	6	Non-pathogenic
13	Fistulina hepatica (Schaeffer: Fr.) Fr	6	Non-pathogenic
14	Octadecabacter sp.	6	Non-pathogenic
15	Octadecabacter sp. ARK10255b	6	Non-pathogenic
16	Halomonas venusta	6	Non-pathogenic
17	Planococcus citreus	6	Non-pathogenic
18	Enterobacter agglomerans	6	Non-pathogenic
19	Aspergillus versicolor	6	Non-pathogenic
20	Streptomyces griseus	6	Non-pathogenic
21	Escherichia sp.	6	Non-pathogenic
22	Bacillus pumilus (BSH-4)	6	Non-pathogenic
23	Burkholderia andropogonis LMG 2129	6	Non-pathogenic
24	Burkholderia sordidicola LMG 22029	6	Non-pathogenic
25	Limnobacter thiooxidans LMG 19593	6	Non-pathogenic
26	Stenotrophomonas rhizophilla ep10-p69	6	Non-pathogenic
27	S.aureus (5)	6	Pathogenic
28	Agaricus bisporus	6	Non-pathogenic
29	Aspergillus candidus	6	Non-pathogenic
30	Emericella nidulans	6	Non-pathogenic
31	Mycoleptodonoides aitchisonii TUFC10099	6	Non-pathogenic
32	P.aeruginosa (2)	6	Pathogenic
33	Pseudomonas fluorescens L13-6-12	6	Non-pathogenic
34	Pseudomonas sp.	6	Non-pathogenic
35	Pseudomonas trivialis 3Re2-7	6	Non-pathogenic
36	Shewanella spp.	6	Non-pathogenic
37	Muscodor fengyangensis (ZJLQ374)	6	Non-pathogenic
38	Penicillium expansum	6	Non-pathogenic
39	Muscodor fengyangensis (ZJLQ070)	6	Non-pathogenic
40	Muscodor fengyangensis (ZJLQ151)	6	Non-pathogenic
41	Muscodor albus I-41. 3s	6	Non-pathogenic

42	Bacillus pumilus (ZB13)	6	Non-pathogenic
43	Bacillus subtilis(BL02)	6	Non-pathogenic
44	Carnobacterium maltaromaticum	6	Non-pathogenic
45	Penicillium crustosum	6	Non-pathogenic
46	E.coli (3)	6	Pathogenic
47	Mycobacterium tuberculosis	6	Pathogenic
48	Psedomonas taetroleus	6	Non-pathogenic
49	Penicillium cyclopium	6	Non-pathogenic
50	Paecilomyces variotii	6	Non-pathogenic
51	Jannaschia helgolandensis strain HEL-26	6	Non-pathogenic
52	Klebsiella pneumoniae	6	Pathogenic
53	Tuber oligospermum	6	Non-pathogenic
54	Thermoactinomyces spp.	6	Non-pathogenic
55	Actinobacteria	6	Non-pathogenic
56	Oscillatoria chalybea	6	Non-pathogenic
57	Oscillatoria sp.	6	Non-pathogenic
58	Streptomyces lavendulae	6	Non-pathogenic
59	Cytophaga strains	6	Non-pathogenic
60	a marine Arctic bacterium	6	Non-pathogenic
61	Burkholderia sordidicola LMG 22029	6	Non-pathogenic
62	Roseobacter clade	6	Non-pathogenic
63	Serratia plymuthica 3Re4-18	6	Non-pathogenic
64	Staphylococcus epidermidis 2P3-18	6	Non-pathogenic
65	Stenotrophomonas rhizophila P69	6	Non-pathogenic
66	Arthrobacter globiformis	6	Non-pathogenic
67	Streptomycete sp.	6	Non-pathogenic
68	Penicillium palitans (commune) (IBT 15899)	6	Non-pathogenic
69	S.aureus (1)	6	Pathogenic
70	Agaricus campestris	6	Non-pathogenic
71	Trichoderma aureoviride IMI 91968	6	Non-pathogenic
72	Aspergillus fumigatus	6	Non-pathogenic
73	Sulfitobacter sp. Bio-007	6	Non-pathogenic
74	Cenococcum geophilum	6	Non-pathogenic
75	Wolinella curva CCUG 13146 (35224)	6	Non-pathogenic
76	aerobic Gram-negative bacteria	6	Non-pathogenic
77	K.pneumoniae (2)	6	Pathogenic
78	S.aureus (2)	6	Pathogenic
79	Trichoderma pseudokoningii (T64)	6	Non-pathogenic
80	Streptomyces àntibioticus CBS 659.68	6	Non-pathogenic
81	psychrotrophic bacteria	6	Non-pathogenic
82	Trichoderma viride (T60)	6	Non-pathogenic
83	Thermomonospora fusca	6	Non-pathogenic
84	Alternaria Alternata	6	Non-pathogenic
85	Paenibacillus polymyxa (BMP-11)	6	Non-pathogenic
86	Puccinia graminis var. tritici	6	Non-pathogenic

87	Trichoderma sp.	6	Non-pathogenic
88	E.coli (2)	6	Pathogenic
89	Bacillus amyloliquefaciens IN937a	6	Non-pathogenic
90	Bacillus subtilis GB03	6	Non-pathogenic
91	Penicillium digitatum	6	Non-pathogenic
92	Bacillus spp.	6	Non-pathogenic
93	Citrobacter freundii	6	Non-pathogenic
94	Micrococcus luteus	6	Non-pathogenic
95	Staphylococcus aureus	6	Pathogenic
96	Daedalea juniperina	6	Non-pathogenic
97	Muscodor fengyangensis (ZJLQ023)	6	Non-pathogenic
98	Muscodor fengyangensis (ZJLQ024)	6	Non-pathogenic
99	Oscillatoria perornata	6	Non-pathogenic
100	Spirulina platensis	6	Non-pathogenic
101	Penicillium clavigerum	6	Non-pathogenic
102	Xanthomonas campestris pv campestris	6	Non-pathogenic
103	Roseovarius spp.	6	Non-pathogenic
104	benthic cyanobacteria (Calothrix, Plectonema)	6	Non-pathogenic
105	Cyanobacterial biofilms	6	Non-pathogenic
106	Aerobasidium pullulans	6	Non-pathogenic
107	E.coli (6)	6	Pathogenic
108	Dipodascus aggregatus	6	Non-pathogenic
109	Pseudomonas solanacearum	6	Non-pathogenic
110	Aspergillus clavatus	6	Non-pathogenic
111	Blastomyces dermatitidis	6	Non-pathogenic
112	Ceratocystis fimbriata	6	Non-pathogenic
113	Mucor hiemalis	6	Non-pathogenic
114	Tuber borchii 43BO	6	Non-pathogenic
115	Tuber borchii ATCC 96540	6	Non-pathogenic
116	Tuber melanosporum Bal1	6	Non-pathogenic
117	Tuber melanosporum Rey_t	6	Non-pathogenic
118	Bifidobacterium adolescentis DPC6044	6	Non-pathogenic
119	Lactobacillus brevis DPC6108	6	Non-pathogenic
120	Anabaena	6	Non-pathogenic
121	Fossombronia pusilla	6	Non-pathogenic
122	Lyngbya	6	Non-pathogenic
123	Sigmatella aurantiaca	6	Non-pathogenic
124	Streptomyces sulfureus	6	Non-pathogenic
125	Streptomyces UC5319	6	Non-pathogenic
126	Pseudomonas fluorescens AN5	6	Non-pathogenic
127	Fomes annosus	6	Non-pathogenic
128	Flavobacteria sp.	6	Non-pathogenic
129	Chromobacterium sp.	6	Non-pathogenic
130	Clitocybe geotropa	6	Non-pathogenic
131	Fomes scutellatus	6	Non-pathogenic

132	Marasmius oreacles	6	Non-pathogenic
133	Pholiota aurea	6	Non-pathogenic
134	Pantoea agglomerans spp.	6	Non-pathogenic
135	Pseudonocardia sp.	6	Non-pathogenic
136	Saccharomonospora sp.	6	Non-pathogenic
137	Thermomonospora sp.	6	Non-pathogenic
138	Lactobacillus fermentum	6	Non-pathogenic
139	Fomes pomaceus	6	Non-pathogenic
140	Alphaproteobacteria (Rhizobium , Sphingomonas , Methylobacterium , Roseovarius)	6	Non-pathogenic
141	Betaproteobacteria (Variovorax , Zogloea)	6	Non-pathogenic
142	Deleya spp.	6	Non-pathogenic
143	Photobacterium spp.	6	Non-pathogenic
144	Plantibacter spp.	6	Non-pathogenic
145	Pseudoalteromonas spp.	6	Non-pathogenic
146	Rhizobium ssp.	6	Non-pathogenic
147	Rhodococcus spp.	6	Non-pathogenic
148	Sphingomonas spp.	6	Non-pathogenic
149	Variovorax spp.	6	Non-pathogenic
150	Vibrio spp.	6	Non-pathogenic
151	Zogloea ssp.	6	Non-pathogenic
152	Bacillus popillae	6	Non-pathogenic
153	Penicillium chrysogenum (IBT 15921)	6	Non-pathogenic
154	Penicillium chrysogenum (IBT 15996)	6	Non-pathogenic
155	Azoarcus evansii	6	Non-pathogenic
156	Acinetobacter calcoaceticus	6	Non-pathogenic
157	Tilletia caries	6	Non-pathogenic
158	Tilletia controversa	6	Non-pathogenic
159	Tilletia foetida	6	Non-pathogenic
160	Bacillus thuringensis	6	Non-pathogenic
161	Chondromyces crocatus Cm c2	7	Non-pathogenic
162	Chondromyces crocatus Cm c5	7	Non-pathogenic
163	Myxobacterium spp.	8	Non-pathogenic
164	Myxococcus xanthus	8	Non-pathogenic
165	Stigmatella aurantiaca	9	Non-pathogenic
166	Stigmatella aurantiaca DW4/3-1	9	Non-pathogenic
167	Stigmatella aurantiaca Sg a15	9	Non-pathogenic
168	Streptomyces caviscabies	10	Non-pathogenic
169	Streptomyces sp. GWS-BW-H5.	10	Non-pathogenic
170	Streptomyces coelicolor	11	Non-pathogenic
171	Streptomyces albidoflavus	11	Non-pathogenic
172	Streptomyces albidoflavus AMI 246	11	Non-pathogenic
173	Streptomyces albus	11	Non-pathogenic
174	Streptomyces albus IFO 13014	11	Non-pathogenic
175	Streptomyces antibioticus	11	Non-pathogenic

176	Streptomyces antibioticus ETH 22014	11	Non-pathogenic
177	Streptomyces aureofaciens ETH 13387	11	Non-pathogenic
178	Streptomyces coelicolor ATCC 21666	11	Non-pathogenic
179	Streptomyces coelicolor DSM 40233	11	Non-pathogenic
180	Streptomyces diastatochromogenes IFO 13814	11	Non-pathogenic
181	Streptomyces griseus ATCC 23345	11	Non-pathogenic
182	Streptomyces griseus IFO 13849	11	Non-pathogenic
183	Streptomyces hirsutus ATCC 19773	11	Non-pathogenic
184	Streptomyces hirsutus ETH 1666	11	Non-pathogenic
185	Streptomyces hygroscopicus ATCC 27438	11	Non-pathogenic
186	Streptomyces murinus DSM 40091	11	Non-pathogenic
187	Streptomyces murinus NRRL 8171	11	Non-pathogenic
188	Streptomyces olivaceus ETH 6445	11	Non-pathogenic
189	Streptomyces olivaceus ETH 7437	11	Non-pathogenic
190	Streptomyces rishiriensis AMI 224	11	Non-pathogenic
191	Streptomyces spp. AMI 240	11	Non-pathogenic
192	Streptomyces spp. AMI 243	11	Non-pathogenic
193	Streptomyces thermoviolaceus CBS 111.62	11	Non-pathogenic
194	Actinomycetes	11	Non-pathogenic
195	Streptomyces albus subsp. pathocidicus IFO 13812	11	Non-pathogenic
196	Streptomyces antibioticus CBS 659.68	11	Non-pathogenic
197	Streptomyces aureofaciens ETH 28832	11	Non-pathogenic
198	Streptomyces diastatochromogenes ETH 18822	11	Non-pathogenic
199	Streptomyces hygroscopicus IFO 13255	11	Non-pathogenic
200	Streptomyces thermoviolaceus IFO 12382	11	Non-pathogenic
201	Streptomyces spp.	12	Non-pathogenic
202	Bacillus	13	Non-pathogenic
203	Tuber borchii	14	Non-pathogenic
204	Tuber indicum	15	Non-pathogenic
205	marine Streptomycete (isolate B6007)	16	Non-pathogenic
206	Prevotella buccae ATCC 33574	16	Non-pathogenic
207	Prevotella buccae ES12-B	16	Non-pathogenic
208	Prevotella buccae ES17-1	16	Non-pathogenic
209	Prevotella buccae ES9-1	16	Non-pathogenic
210	Prevotella disiens DSM 20516	16	Non-pathogenic
211	Prevotella heparinolyticus ATCC 35895	16	Non-pathogenic
212	Prevotella oris ATCC 33573	16	Non-pathogenic
213	Prevotella oris ES14B-3A	16	Non-pathogenic
214	Prevotella oris ES9-3	16	Non-pathogenic
215	Prevotella oris RPG	16	Non-pathogenic
216	Prevotella veroralis ATCC 33779	16	Non-pathogenic
217	Porphyromonas endodontalis HG 181 (H 11a-e)	16	Non-pathogenic
218	Porphyromonas endodontalis HG 182 (BN 11a-f)	16	Non-pathogenic
219	Porphyromonas endodontalis HG 370 (ATCC 35406)	16	Non-pathogenic
220	Porphyromonas endodontalis HG 412	16	Non-pathogenic

221	Prevotella oralis ES4-B	16	Non-pathogenic
222	Bacteroides fragilis	16	Non-pathogenic
223	Bacteroides fragilis ATCC 25285	16	Non-pathogenic
224	Prevotella oralis ES14B-3A	16	Non-pathogenic
225	Prevotella oralis ES15-2	16	Non-pathogenic
226	ARK10063	17	Non-pathogenic
227	Bjerkandera adusta	17	Non-pathogenic
228	Bjerkandera adusta CBS 595.78	17	Non-pathogenic
229	Armillaria mellea	18	Non-pathogenic
230	Pholiota squarrosa	18	Non-pathogenic
231	Stropharia rugosoannulata	18	Non-pathogenic
232	Verticillium longisporum	19	Non-pathogenic
233	Candida tropicalis	19	Non-pathogenic
234	Salmonella enterica	19	Non-pathogenic
235	Shigella flexneri	19	Non-pathogenic
236	Tuber panniferum	19	Non-pathogenic
237	Tuber excavatum	19	Non-pathogenic
238	Penicillium aurantiogriseum	19	Non-pathogenic
239	Ascocoryne sarcoides NRRL 50072	19	Non-pathogenic
240	Aspergillus ornatus	19	Non-pathogenic
241	Neurospora sitophila ATCC 46892	19	Non-pathogenic
242	Neurospora sp.	19	Non-pathogenic
243	Penicillium chrysogenum	19	Non-pathogenic
244	penicillium paneum (Conidia)	19	Non-pathogenic
245	Tuber uncinatum	19	Non-pathogenic
246	Ceratocystis sp.	19	Non-pathogenic
247	Thielaviopsis basicola	19	Non-pathogenic
248	Mycobacterium bovis	19	Non-pathogenic
249	Muscodor albus CZ-620	19	Non-pathogenic
250	Muscodor crispans	19	Non-pathogenic
251	Boletus variegatus	19	Non-pathogenic
252	Fomes sp.	19	Non-pathogenic
253	Dinoroseobacter shibae	20	Non-pathogenic
254	Dinoroseobacter shibae strain DFL-27	20	Non-pathogenic
255	Loktanella sp.	20	Non-pathogenic
256	Loktanella sp. Bio-204	20	Non-pathogenic
257	Carnobacterium divergens 9P	21	Non-pathogenic
258	Dinoroseobacter sp.	22	Non-pathogenic
259	Stigmatella sp.	22	Non-pathogenic
260	Calothrix	23	Non-pathogenic
261	Phormidium sp.	23	Non-pathogenic
262	Plectonema	23	Non-pathogenic
263	Calothrix parietina	23	Non-pathogenic
264	Plectonema notatum	23	Non-pathogenic
265	Plectonema sp.	23	Non-pathogenic

266	Tolypothrix	23	Non-pathogenic
267	Tolypothrix distorta	23	Non-pathogenic
268	Calothrix sp.	23	Non-pathogenic
269	Burkholderia ambifaria LMG 19467	24	Non-pathogenic
270	Burkholderia ambifaria LMG 17828	24	Non-pathogenic
271	Burkholderia ambifaria LMG 19182	24	Non-pathogenic
272	Alcaligenes	25	Non-pathogenic
273	Alcaligenes faecalis	25	Non-pathogenic
274	Arthrobacter nitroguajacolius	25	Non-pathogenic
275	Lysobacter gummosus	25	Non-pathogenic
276	Sporosarcina ginsengisoli	25	Non-pathogenic
277	Stenotrophomonas maltophilia	26	Non-pathogenic
278	Serratia marcescens	26	Non-pathogenic
279	Bacillus simplex	26	Non-pathogenic
280	Bacillus subtilis	26	Non-pathogenic
281	Bacillus weihenstephanensis	26	Non-pathogenic
282	Microbacterium oxydans	26	Non-pathogenic
283	Streptomyces lateritius	26	Non-pathogenic
284	Escherichia coli	27	Pathogenic
285	Burkholderia anthina LMG 20980	27	Non-pathogenic
286	Burkholderia gladioli LMG 2216	27	Non-pathogenic
287	Burkholderia glumae LMG 2196	27	Non-pathogenic
288	Burkholderia caledonica LMG 19076	27	Non-pathogenic
289	Burkholderia caribensis LMG 18531	27	Non-pathogenic
290	Burkholderia caryophylli LMG 2155	27	Non-pathogenic
291	Burkholderia fungorum LMG 16225	27	Non-pathogenic
292	Burkholderia glathei LMG 14190	27	Non-pathogenic
293	Burkholderia lata LMG 22485	27	Non-pathogenic
294	Serratia plymuthica IC14	27	Non-pathogenic
295	Burkholderia graminis LMG 18924	27	Non-pathogenic
296	Cellulomonas uda	27	Non-pathogenic
297	Paenibacillus polymyxa	28	Non-pathogenic
298	Paenibacillus polymyxa E681	28	Non-pathogenic
299	Trichoderma viride	29	Non-pathogenic
300	Tuber aestivum	30	Non-pathogenic
301	Tuber brumale	31	Non-pathogenic
302	Tuber mesentericum	31	Non-pathogenic
303	Tuber rufum	31	Non-pathogenic
304	Tuber simonea	31	Non-pathogenic
305	Pseudomonas fragi 25P	32	Non-pathogenic
306	Burkholderia lata LMG 6993	33	Non-pathogenic
307	Burkholderia phenazinium LMG 2247	33	Non-pathogenic
308	Burkholderia phytofirmans LMG 22487	33	Non-pathogenic
309	Burkholderia pyrrocinia LMG 21822	33	Non-pathogenic
310	Burkholderia terricola LMG 20594	33	Non-pathogenic

311	Chromobacterium violaceum	33	Non-pathogenic
312	Chromobacterium violaceum CV0	33	Non-pathogenic
313	Pseudomonas putida	33	Non-pathogenic
314	Pseudomonas putida ISOf	33	Non-pathogenic
315	Serratia marcescens MG1	33	Non-pathogenic
316	Serratia plymuthica HRO-C48	33	Non-pathogenic
317	Burkholderia sacchari LMG 19450	33	Non-pathogenic
318	Burkholderia thailandensis LMG 20219	33	Non-pathogenic
319	Pseudomonas fluorescens WCS 417r	33	Non-pathogenic
320	Serratia entomophilia A1MO2	33	Non-pathogenic
321	Serratia proteamaculans B5a	33	Non-pathogenic
322	Burkholderia tropica LMG 22274	34	Non-pathogenic
323	Burkholderia cepacia LMG 1222 358	34	Non-pathogenic
324	Burkholderia hospita LMG 20598	34	Non-pathogenic
325	Burkholderia kururiensis LMG 19447	34	Non-pathogenic
326	Burkholderia phenoliruptrix LMG 22037	34	Non-pathogenic
327	Burkholderia xenovorans LMG 21463	34	Non-pathogenic
328	Serratia sp.	34	Non-pathogenic
329	Pandoraea norimbergensis LMG 18379	34	Non-pathogenic
330	E.coli (1)	35	Pathogenic
331	E.coli (4)	35	Pathogenic
332	K.pneumoniae (1)	35	Pathogenic
333	K.pneumoniae (3)	35	Pathogenic
334	E.coli (7)	35	Pathogenic
335	E.coli (8)	35	Pathogenic
336	E.coli (9)	35	Pathogenic
337	E.coli (10)	35	Pathogenic
338	E.coli (11)	35	Pathogenic
339	E.coli (12)	35	Pathogenic
340	E.coli (13)	35	Pathogenic
341	E.coli (14)	35	Pathogenic
342	E.coli (15)	35	Pathogenic
343	E.coli (16)	35	Pathogenic
344	E.coli (17)	35	Pathogenic
345	E.coli (18)	35	Pathogenic
346	E.coli (19)	35	Pathogenic
347	E.coli (20)	35	Pathogenic
348	E.coli (21)	35	Pathogenic
349	E.coli (22)	35	Pathogenic
350	E.coli (23)	35	Pathogenic
351	E.coli (24)	35	Pathogenic
352	K.pneumoniae (4)	35	Pathogenic
353	K.pneumoniae (5)	35	Pathogenic
354	K.pneumoniae (6)	35	Pathogenic
355	K.pneumoniae (7)	35	Pathogenic

356	K.pneumoniae (8)	35	Pathogenic
357	K.pneumoniae (9)	35	Pathogenic
358	K.pneumoniae (15)	35	Pathogenic
359	K.pneumoniae (16)	35	Pathogenic
360	K.pneumoniae (17)	35	Pathogenic
361	K.pneumoniae (18)	35	Pathogenic
362	P.aeruginosa (1)	35	Pathogenic
363	K.pneumoniae (13)	35	Pathogenic
364	K.pneumoniae (14)	35	Pathogenic
365	Pseudomonas aurantiaca	36	Non-pathogenic
366	Pseudomonas chlororaphis	36	Non-pathogenic
367	Pseudomonas corrugate	36	Non-pathogenic
368	Pseudomonas fluorescens	36	Non-pathogenic
369	Cupriavidus necator LMG 1199	37	Non-pathogenic
370	Klebsiella sp.	37	Non-pathogenic
371	Pseudomonas aeruginosa PUPa3	37	Non-pathogenic
372	Citrobacter sp.	37	Non-pathogenic
373	Enterobacter spp.	37	Non-pathogenic
374	Lactobacillus brevis	37	Non-pathogenic
375	Lactobacillus hilgardii	37	Non-pathogenic
376	Oenococcus oeni	37	Non-pathogenic
377	Lactobacillus lactis	37	Non-pathogenic
378	Alpha proteobacteria	37	Non-pathogenic
379	Gamma proteobacteria	37	Non-pathogenic
380	Klebsiella oxytoca	37	Non-pathogenic
381	Lactobacillus sp.	37	Non-pathogenic
382	Lactococcus sp.	37	Non-pathogenic
383	Schizophyllum commune	37	Non-pathogenic
384	Alphaproteobacteria (e.g. Roseobacter sp.)	37	Non-pathogenic
385	Betaproteobacteria (Alcaligenes faecalis)	37	Non-pathogenic
386	Desulfovibrio acrylicus	37	Non-pathogenic
387	Parasporobacterium paucivorans	37	Non-pathogenic
388	Treponema denticola	37	Non-pathogenic
389	Brevibacterium linens	37	Non-pathogenic
390	Aspergillus flavus	38	Non-pathogenic
391	Aspergillus flavus NRRL 18543	38	Non-pathogenic
392	Aspergillus flavus NRRL 25347	38	Non-pathogenic
393	Aspergillus niger	38	Non-pathogenic
394	Aspergillus niger NRRL 326	38	Non-pathogenic
395	Aspergillus parasiticus NRRL 5862	38	Non-pathogenic
396	Penicillium glabrum NRRL 766	38	Non-pathogenic
397	Rhizopus stolonifer	38	Non-pathogenic
398	Rhizopus stolonifer NRRL 54667	38	Non-pathogenic
399	Serratia proteamaculans 42M	39	Non-pathogenic
400	E. cloacae	40	Pathogenic

401	S.aureus (3)	40	Pathogenic
402	K.pneumoniae (11)	40	Pathogenic
403	S.aureus (6)	40	Pathogenic
404	S.aureus (8)	40	Pathogenic
405	S.aureus (10)	40	Pathogenic
406	S.aureus (11)	40	Pathogenic
407	S.aureus (13)	40	Pathogenic
408	S.aureus (14)	40	Pathogenic
409	S.aureus (17)	40	Pathogenic
410	S.aureus (20)	40	Pathogenic
411	K.pneumoniae (10)	40	Pathogenic
412	K.pneumoniae (12)	40	Pathogenic
413	K.pneumoniae (19)	40	Pathogenic
414	K.pneumoniae (20)	40	Pathogenic
415	S.aureus (7)	40	Pathogenic
416	S.aureus (9)	40	Pathogenic
417	S.aureus (12)	40	Pathogenic
418	S.aureus (15)	40	Pathogenic
419	S.aureus (16)	40	Pathogenic
420	S.aureus (18)	40	Pathogenic
421	S.aureus (19)	40	Pathogenic
422	S.aureus (21)	40	Pathogenic
423	S.aureus (22)	40	Pathogenic
424	biofilms A (Rivularia sp./C. parietina community)	41	Non-pathogenic
425	C. parietina	41	Non-pathogenic
426	Cyanobacteria	41	Non-pathogenic
427	Rivularia sp.	41	Non-pathogenic
428	Laccaria bicolor	42	Non-pathogenic
429	Paxillus involutus MAJ	42	Non-pathogenic
430	Paxillus involutus NAU	42	Non-pathogenic
431	Penicillium sp.	43	Non-pathogenic
432	Desulfovibrio gigas	43	Non-pathogenic
433	Methanobacterium formicicum	43	Non-pathogenic
434	Methanobacterium thermoautotrophicum	43	Non-pathogenic
435	Methanosarcina barkeri	43	Non-pathogenic
436	Aeromonas veronii	43	Non-pathogenic
437	Geobacillus stearothermophilus	43	Non-pathogenic
438	Clostridium collagenovorans	43	Non-pathogenic
439	Desulfovibrio vulgaris	43	Non-pathogenic
440	Enterobacter cloacae	43	Non-pathogenic
441	Rhodobacter spaeroides	43	Non-pathogenic
442	Rhodocyclus tenuis	43	Non-pathogenic
443	Rhodospirillum rubrum	43	Non-pathogenic
444	Aspergillus sp.	43	Non-pathogenic
445	Candida humicola	43	Non-pathogenic

446	Scopulariopsis brevicaulis	43	Non-pathogenic
447	Methanobacterium sp.	43	Non-pathogenic
448	Bacterium from CFB group	43	Non-pathogenic
449	Saccharomyces cerevisiae Y1001	44	Non-pathogenic
450	Serratia spp. B2675	44	Non-pathogenic
451	Serratia spp. B675	44	Non-pathogenic
452	Saccharomyces cerevisiae	44	Non-pathogenic
453	Xanthomonas campestris pv. vesicatoria 85-10	45	Non-pathogenic
454	Sulfitobacter pontiacus	46	Non-pathogenic
455	Sulfitobacter pontiacus BIO-007	46	Non-pathogenic
456	Sulfitobacter sp.	46	Non-pathogenic
457	Loktanella hongkongensis strain Bio-204	46	Non-pathogenic
458	Sulfitobacter dubius BIO-205	46	Non-pathogenic
459	bacterial strains from the North Sea, the Arctic Ocean, or of terrestrial origin	46	Non-pathogenic
460	Oceanibulbus indolifex HEL-45	46	Non-pathogenic
461	Roseobacter gallaeciensis strain PIC-68	46	Non-pathogenic
462	Stappia marina strain DFL-11	46	Non-pathogenic
463	Sulfitobacter sp. PIC-70	46	Non-pathogenic
464	E.coli (5)	47	Pathogenic
465	S.aureus (4)	47	Pathogenic
466	Staphylococcus sp.	48	Pathogenic
467	Staphylococcus xylosus	48	Pathogenic
468	Clostridium sp.	48	Pathogenic
469	Bacteroides distasonis	48	Non-pathogenic
470	Bacteroides ovatus	48	Non-pathogenic
471	Bacteroides thetaiotamicron	48	Non-pathogenic
472	Bacteroides vulgatus	48	Non-pathogenic
473	Capnocytophaga ochracea ATCC 33596	48	Non-pathogenic
474	Clostridium bifermentans	48	Non-pathogenic
475	Clostridium sporogenes	48	Non-pathogenic
476	Fusobacterium nucleatum	48	Non-pathogenic
477	Porphyromonas gingivalis	48	Non-pathogenic
478	Porphyromonas gingivalis FDC381	48	Non-pathogenic
479	Porphyromonas gingivalis W83	48	Pathogenic
480	Prevotella intermedia ATCC 25261	48	Non-pathogenic
481	Prevotella loescheii	48	Non-pathogenic
482	Prevotella loescheii ATCC 15930	48	Non-pathogenic
483	Veillonella spp.	48	Non-pathogenic
484	Actinobacillus actinomycetemcomitans Y4	48	Non-pathogenic
485	Bacteroides bivius	48	Non-pathogenic
486	Clostridium butyricum	48	Non-pathogenic
487	Clostridium cadaverum	48	Non-pathogenic
488	Clostridium fallax	48	Non-pathogenic
489	Clostridium histolyticum	48	Non-pathogenic
490	Clostridium tertium	48	Non-pathogenic

491	Lactobacillus casei NCIB 8010	49	Non-pathogenic
492	Lactobacillus plantarum	49	Non-pathogenic
493	Lactobacillus plantarum NCIB 6376	49	Non-pathogenic
494	Lactococcus lactis	49	Non-pathogenic
495	Lactococcus lactis DSM 20202	49	Non-pathogenic
496	Leuconostoc cremoris DSM 20346	49	Non-pathogenic
497	Leuconostoc dextranicum DSM 20484	49	Non-pathogenic
498	Leuconostoc mesenteroides DSM 20343	49	Non-pathogenic
499	Leuconostoc oenos	49	Non-pathogenic
500	Leuconostoc oenos B66	49	Non-pathogenic
501	Leuconostoc oenos 19	49	Non-pathogenic
502	Leuconostoc oenos 30	49	Non-pathogenic
503	Leuconostoc oenos 36	49	Non-pathogenic
504	Leuconostoc oenos 37D	49	Non-pathogenic
505	Leuconostoc oenos 7B	49	Non-pathogenic
506	Leuconostoc oenos DSM 20252	49	Non-pathogenic
507	Leuconostoc oenos DSM 20255	49	Non-pathogenic
508	Leuconostoc oenos DSM 20257	49	Non-pathogenic
509	Leuconostoc oenos Lc5x	49	Non-pathogenic
510	Leuconostoc paramesenteroides DSM 20288	49	Non-pathogenic
511	Pediococcus damnosus DSM 20331	49	Non-pathogenic
512	Bacteroides gracilis CCUG 13143 (ATCC 33236)	50	Non-pathogenic
513	Bacteroides ureolyticus CCUG 7319 (ATCC 33387)	50	Non-pathogenic
514	Campylobacter fetus subsp. venerealis CCUG 538 (ATCC 19438)	50	Non-pathogenic
515	Wolinella recta FDC 371 (ATCC 33238)	50	Non-pathogenic
516	Wolinella succinogenes CCUG 12550 (ATCC 29543)	50	Non-pathogenic
517	Wolinella curva CCUG 13146 (ATCC 35224)	50	Non-pathogenic

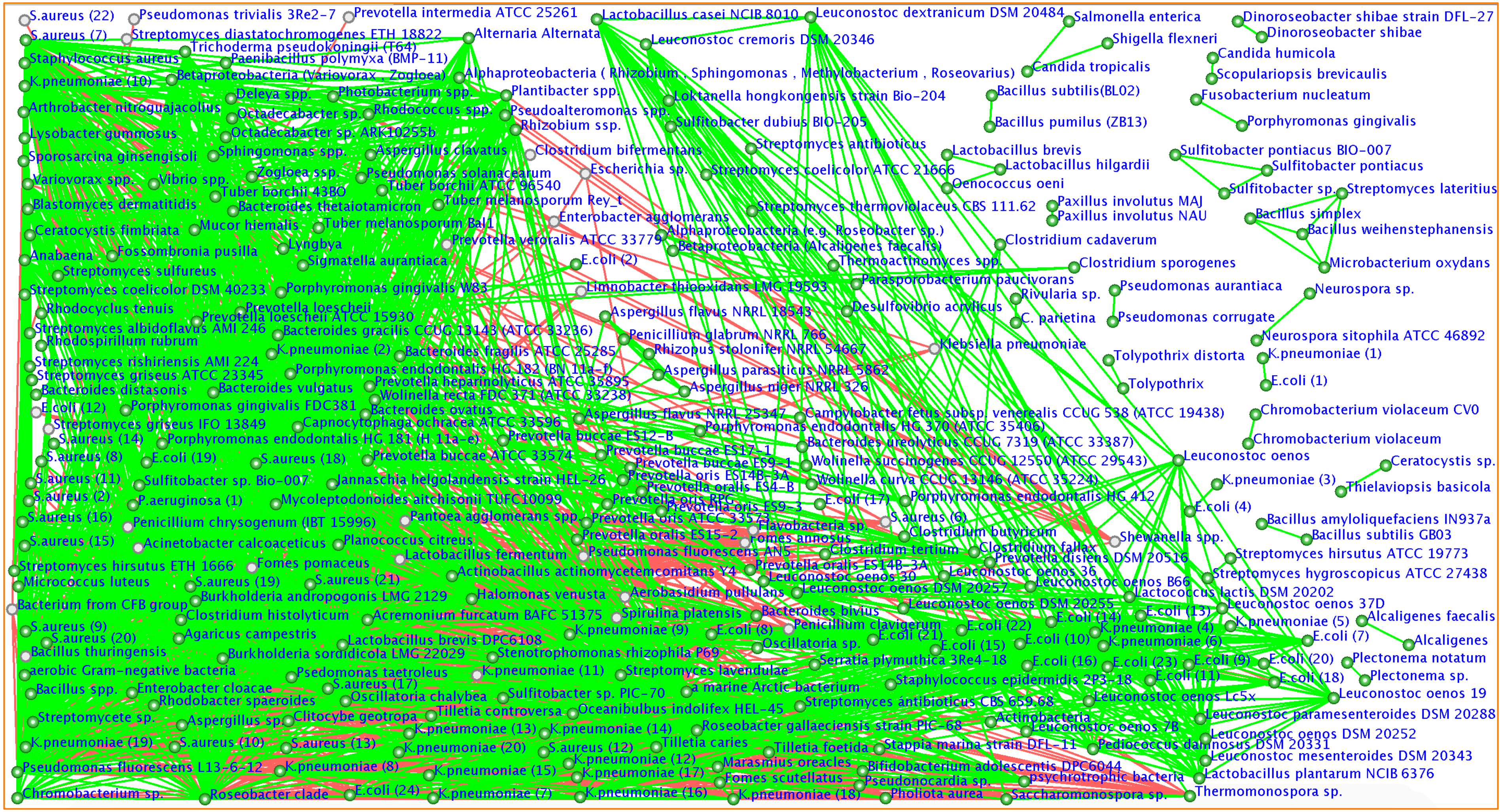

Supplementary Figure 1

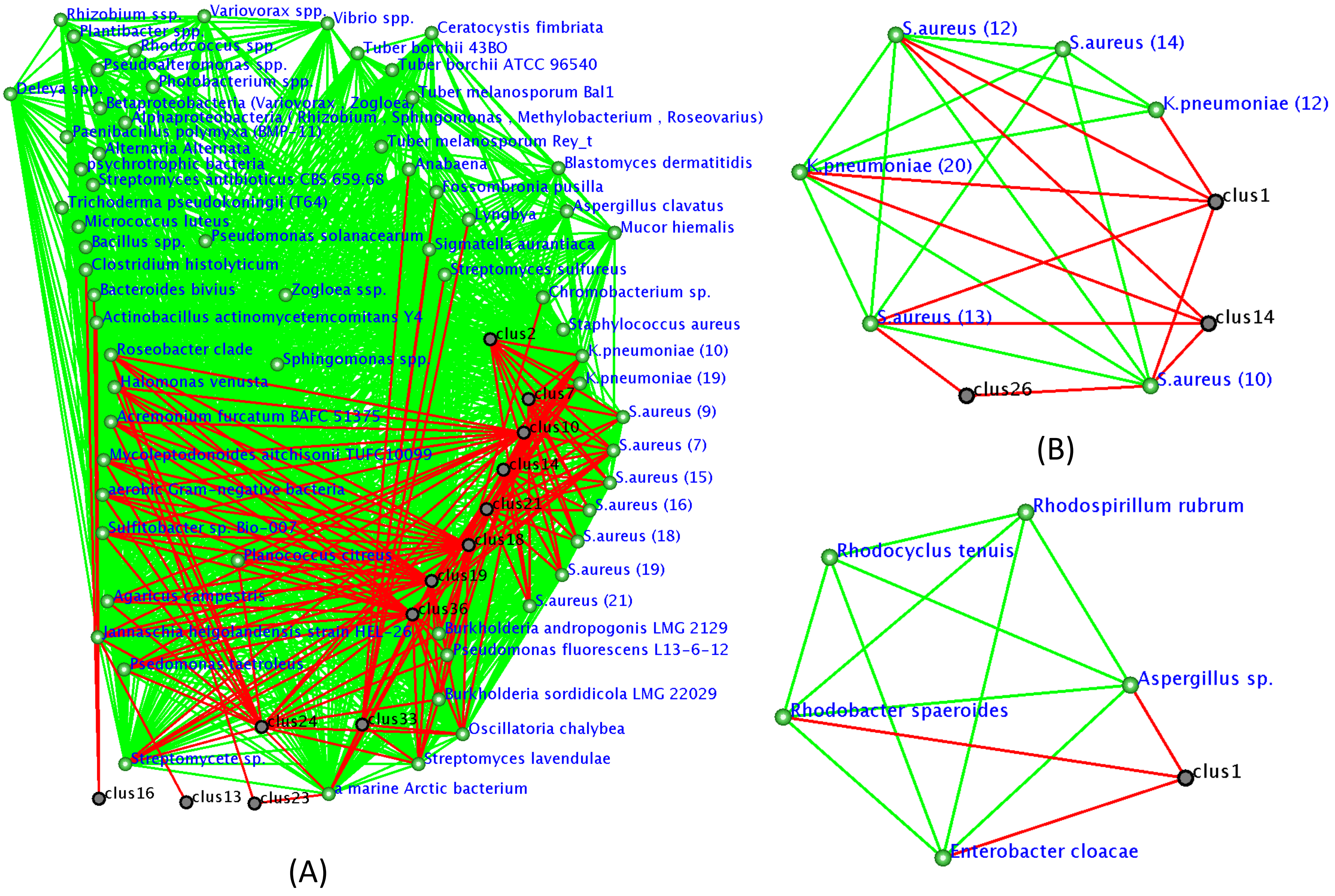

Supplementary Figure 2

Cluster 1 (55 VOCs)

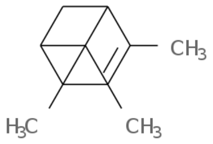

C00000805
alpha-Pinene
PubChem CID: 6654
C10H16

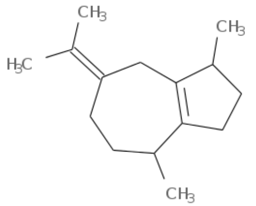

C00020376
beta-Guaiene
PubChem CID: 6949
C15H24

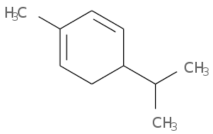

C00003051
alpha-Phellandrene
PubChem CID: 7460
C10H16

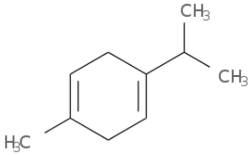

gamma-Terpinen
PubChem CID: 7461
C10H16

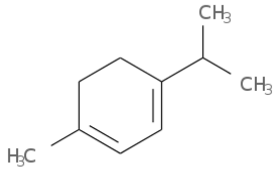

C00003060
alpha-Terpinene
PubChem CID: 7462
C10H16

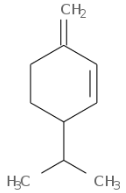

C00010872
abeta-Phellandrene
PubChem CID: 11142
C10H16

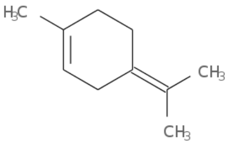

Terpinolene
PubChem CID: 11463
C10H16

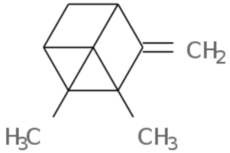

C00000816
beta-Pinene
PubChem CID: 14896
C10H16

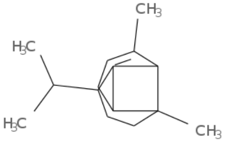

C00003118
Copaene
PubChem CID: 19725
C15H24

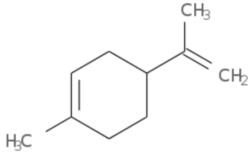

C00010868
d-Limonene
PubChem CID: 22311
C10H16

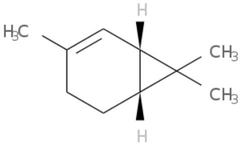

(+)-2-Carene
PubChem CID: 78249
C10H16

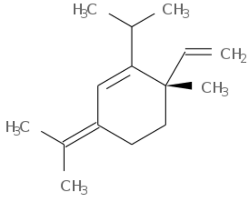

a-Elemene
PubChem CID: 80048
C15H24

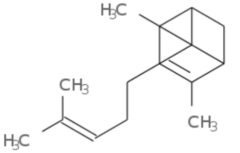

(E)-alpha-Bergamotene
PubChem CID: 86608
C15H24

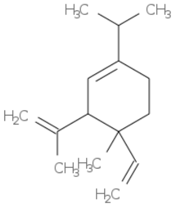

C00012011
delta-Elemene
PubChem CID: 89316
C15H24

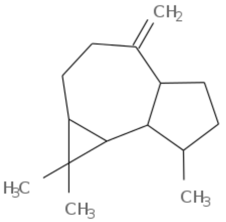

C00021230
Aromadendrene
PubChem CID: 91354
C15H24

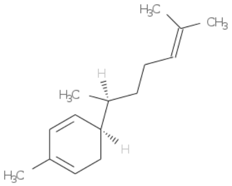

C00003204
alpha-Zingiberene
PubChem CID: 92776
C15H24

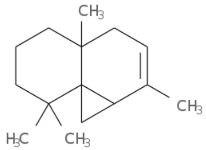

C00003194
Thujopsene
PubChem CID: 97829
C15H24

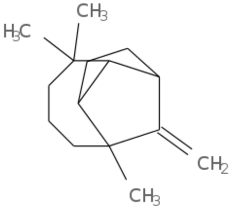

C00003162
Longifolene
PubChem CID: 289151
C15H24

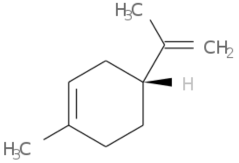

(+)-Limonene
PubChem CID: 440917
C10H16

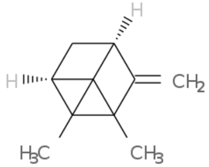

C00000806
(-)-beta-Pinene
PubChem CID: 440967
C10H16

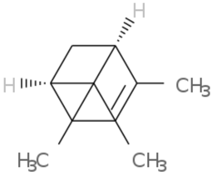

1-alpha-Pinene
PubChem CID: 440968
C10H16

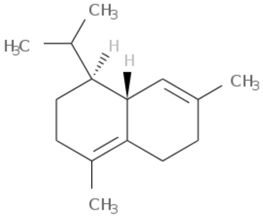

C00007636
delta-Cadinene
PubChem CID: 441005
C15H24

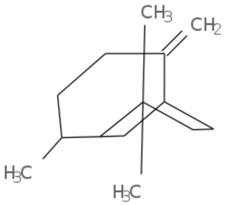

C00021999
Seychellene
PubChem CID: 519743
C15H24

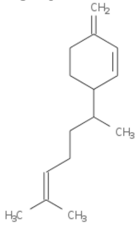

Sesquiphellandrene
PubChem CID: 519764
C15H24

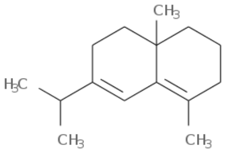

C00007634
delta-Selinene
PubChem CID: 520383
C15H24

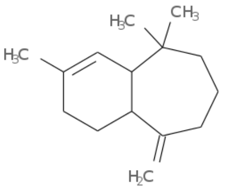

C00021309
alpha-Himachalene
PubChem CID: 520909
C15H24

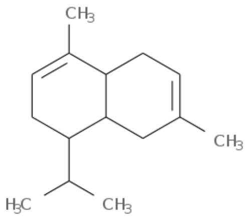

Cadinene
PubChem CID: 521380
C15H24

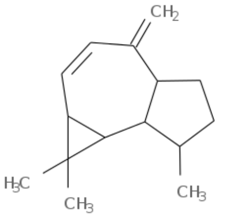

Dehydroaromadendrene
PubChem CID: 526687
C15H22

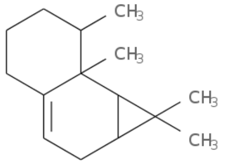

C00017471
Aristolene
PubChem CID: 530421
C15H24

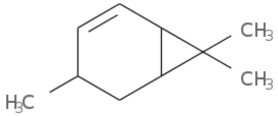

(+)-4-Carene
PubChem CID: 530422
C10H16

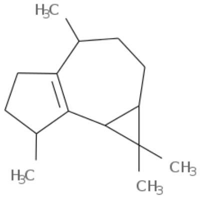

C00037332
Isoledene
PubChem CID: 530426
C15H24

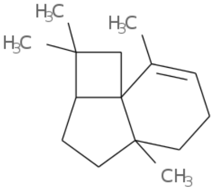

alpha-Panasinsen
PubChem CID: 578929
C15H24

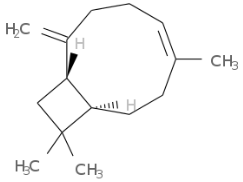

C00003110
beta-Caryophyllene
PubChem CID: 5281515
C15H24

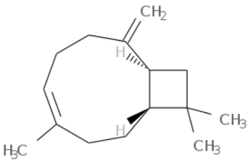

C00012474
Isocaryophyllene
PubChem CID: 5281522
C15H24

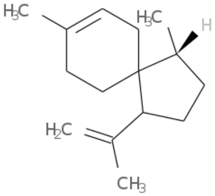

C00021580
beta-Acoradiene
PubChem CID: 5316209
C15H24

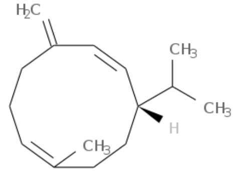

(-)-Germacrene D
PubChem CID: 5317570
C15H24

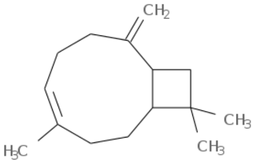

C00003110
Caryophyllene
PubChem CID: 5322111
C15H24

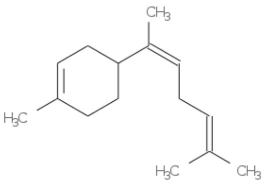

alpha-Bisabolene
PubChem CID: 5352653
C15H24

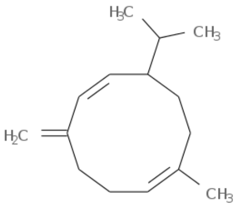

C00011720
Caryophyllene
PubChem CID: 5373727
C15H24

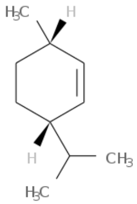

2-Menthene
PubChem CID: 6427082
C10H18

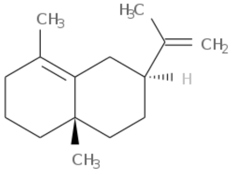

4,11-Selinadiene
PubChem CID: 6429320
C15H24

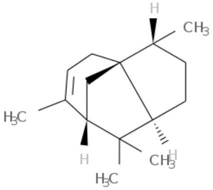

C00003111
alpha-Cedrene
PubChem CID: 6431015
C15H24

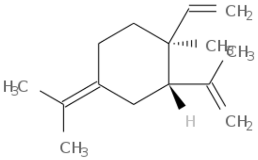

C00012012
gamma-Elemene
PubChem CID:6432312
C15H24

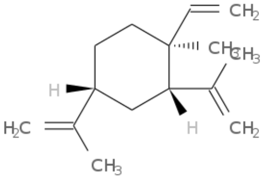

C00007453
beta-Elemene
PubChem CID: 6918391
C15H24

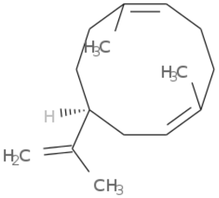

C00011719
Germacrene A
PubChem CID:9548706
C15H24

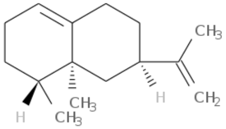

C00034741
Valencene
PubChem CID: 9855795
C15H24

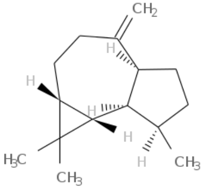

C00021229
Alloaromadendrene
PubChem CID: 10899740
C15H24

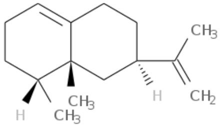

C00016975
Eremophilene
PubChem CID: 11160025
C15H24

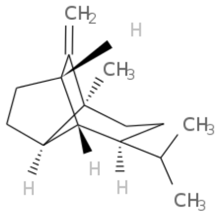

(+)-Sativene
PubChem CID: 11275742
C15H24

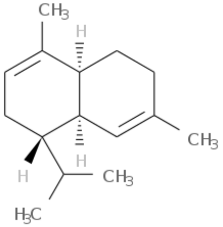

C00029671
alpha-Muurolene
PubChem CID: 12306047
C15H24

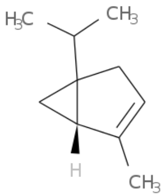

C00000184
alpha-Thujene
PubChem CID: 12444322
C10H16

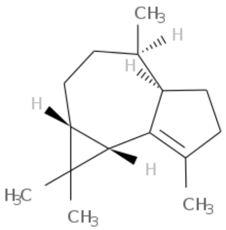

C00021227
alpha-Gurjunene
PubChem CID: 15560276
C15H24

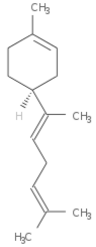

C00050259
Trans-alpha-bisabolene
PubChem CID: 24798703
C15H24

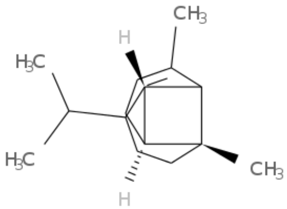

C00003118
alpha-Copaene
PubChem CID: 25245021
C15H24

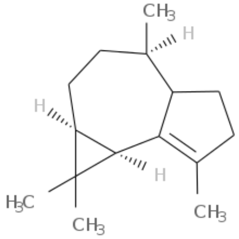

Gurjunene
PubChem CID: 57369711
C15H24

Cluster 2 (33 VOCs)

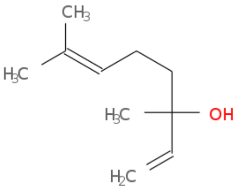

beta-Linalool
PubChem CID: 6549
C10H18O

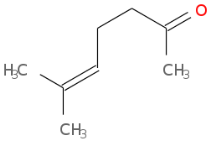

C00034775
6-Methyl-5-hepten-2-one
PubChem CID: 9862
C8H14O

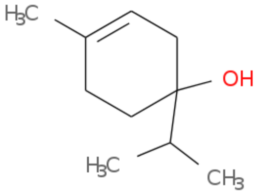

C00029544
Terpinen-4-ol
PubChem CID: 11230
C10H18O

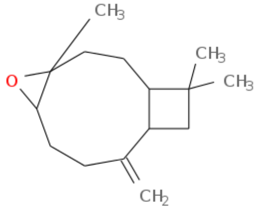

C00012483
Caryophyllene oxide
PubChem CID: 14350
C15H24O

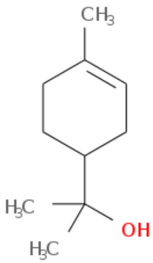

p-Menth-1-en-8-ol
PubChem CID: 17100
C15H24O

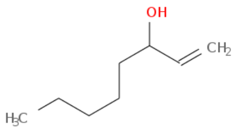

C00029423
1-Octen-3-ol
PubChem CID: 18827
C8H16O

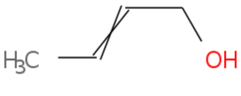

2-Buten-1-ol
PubChem CID: 20024
C4H8O

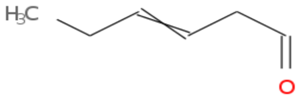

C00048948
3-Hexenal
PubChem CID:23234
C6H10O

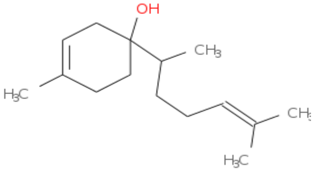

beta-Bisobolol
PubChem CID: 27208
C15H26O

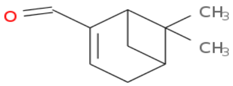

C00030803
Myrtenal
PubChem CID:61130
C10H14O

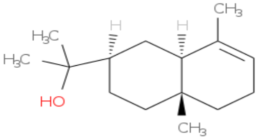

C00000163
alpha-Eudesmol
PubChem CID:92762
C15H26O

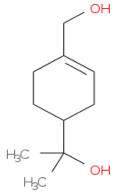

p-Menth-1-en-8-ol
PubChem CID: 110662
C10H18O2

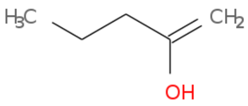

1-Penten-2-on
PubChem CID: 173840
C5H10O

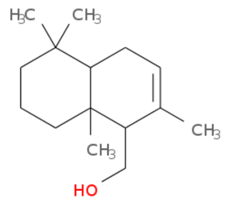

C00020282
Drimenol
PubChem CID: 298071
C15H26O

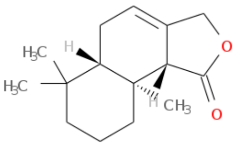

C00003252
Drimenin
PubChem CID: 442202
C15H22O2

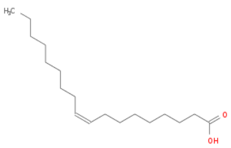

C00001232
Oleic acid
PubChem CID: 445639
C18H34O2

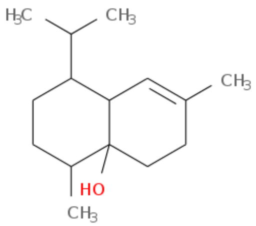

C00033734
Cubenol
PubChem CID: 519857
C15H26O

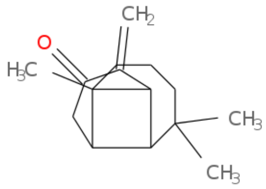

Longipinocarvone
PubChem CID: 535296
C15H22O

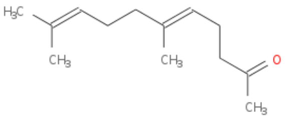

Geranylacetone
PubChem CID: 1549778
C13H22O

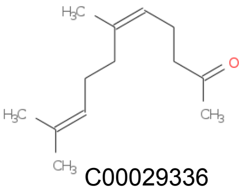

C00029336
(E)-Geranyl acetone
PubChem CID: 1713001
C13H22O

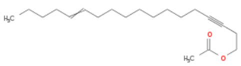

Z-3-Octadecen-1-ol acetate
PubChem CID: 3085631
C20H34O2

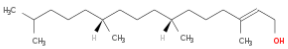

C00003467
Phytol
PubChem CID: 5280435
C20H40O

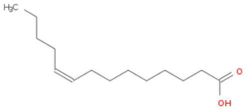

C00001229
Myristoleic acid
PubChem CID: 5281119
C14H26O2

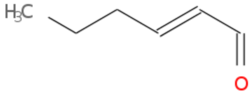

(E)-2-Hexenal
PubChem CID: 5281168
C6H10O

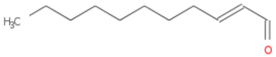

2-Undecenal
PubChem CID: 5283356
C11H20O

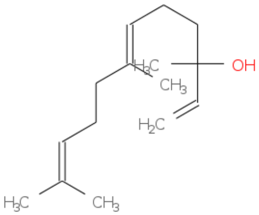

C00003166
Nerolidol
PubChem CID: 5284507
C15H26O

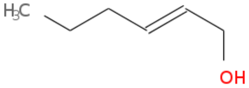

2-Hexenol
PubChem CID: 5318042
C6H12O

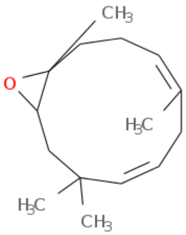

C00012443
Humulene epoxide
PubChem CID: 5352470
C15H24O

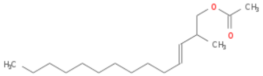

E-2-Methyl-3-tetradecen-1-ol acetate
PubChem CID: 5363512
C17H32O2

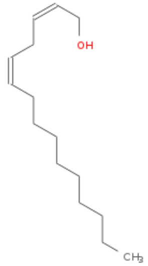

Z,Z-2,5-Pentadecadien-1-ol
PubChem CID: 5364952
C8H16O

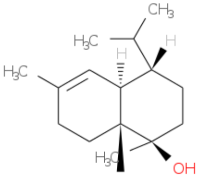

C00020065
alpha-Cadinol
PubChem CID: 10398656
C15H26O

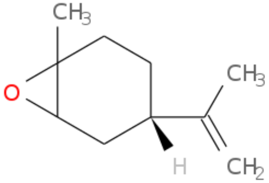

C00035852
Limonene oxide
PubChem CID: 10953718
C10H16O

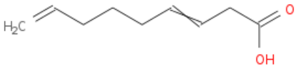

(2E,6E)-2,6-Nonadienoic acid
PubChem CID: 54098240
C9H14O2

Cluster 3 (41 VOCs)

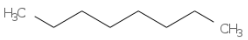

C00035857
Octane
PubChem CID: 356
C8H18

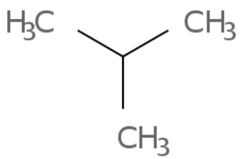

Isobutane
PubChem CID: 6360
C4H10

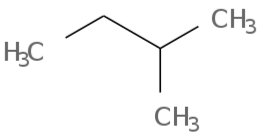

2-Methyl-butane
PubChem CID: 6556
C5H12

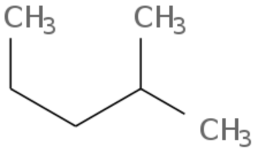

2-Methylpentane
PubChem CID: 7892
C6H14

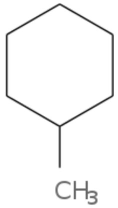

C00035853
Methylcyclohexane
PubChem CID: 7962
C7H14

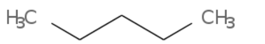

Pentane
PubChem CID: 8003
C5H12

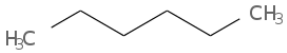

Hexane
PubChem CID: 8058
C6H14

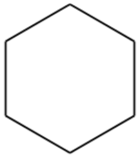

C00007453
Cyclohexane
PubChem CID: 8078
C6H12

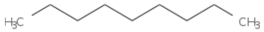

C00034882
Nonane
PubChem CID: 8141
C9H20

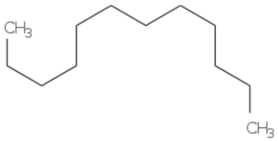

C00001248
Dodecane
PubChem CID: 8182
C12H26

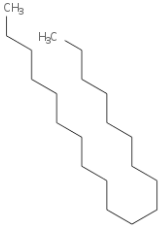

C00030165
Eicosane
PubChem CID: 8222
C20H42

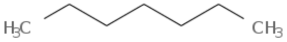

Heptane
PubChem CID: 8900
C7H16

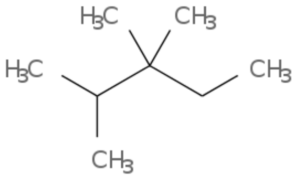

2,3,3-Trimethylpentane
PubChem CID: 11215
C8H18

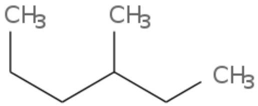

C00050708
3-Methylhexane
PubChem CID: 11507
C7H16

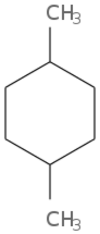

1,4-Dimethylcyclohexane
PubChem CID: 11523
C8H16

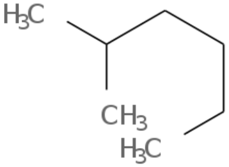

C00035484
2-Methylhexane
PubChem CID: 11582
C7H16

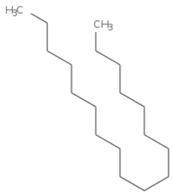

C00030879
Octadecane
PubChem CID: 11635
C18H38

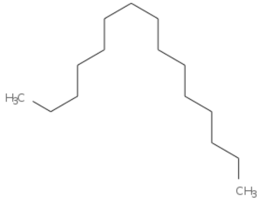

C00001265
Pentadecane
PubChem CID: 12391
C15H32

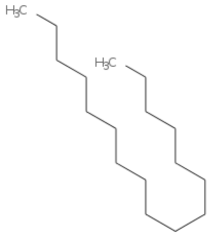

C00030472
Heptadecane
PubChem CID: 12398
C17H36

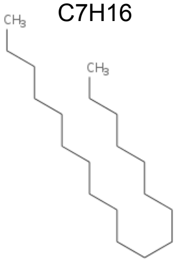

C00030827
Nonadecane
PubChem CID: 12401
C19H40

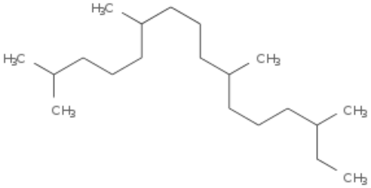

Phytane
PubChem CID: 12523
C20H42

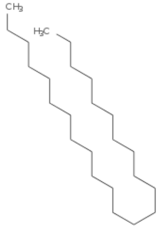

C00032307
Tetracosane
PubChem CID: 12592
C24H50

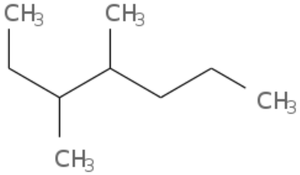

2,4-Dimethylheptane
PubChem CID: 13534
C9H20

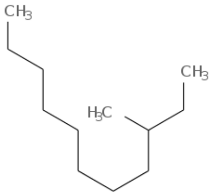

3-Methylundecane
PubChem CID: 13845
C12H26

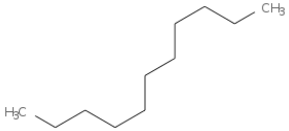

C00032443
Undecane
PubChem CID: 14257
C11H24

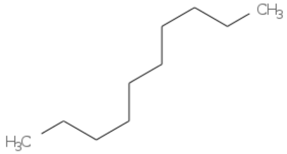

C00048375
Decane
PubChem CID: 15600
C10H22

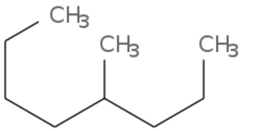

4-Methyloctane
PubChem CID: 16665
C9H20

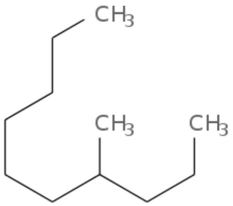

4-Methyl decane
PubChem CID: 17835
C11H24

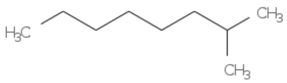

2-Methyloctane
PubChem CID: 18591
C9H20

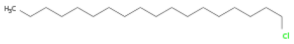

1-Chlorooctadecane
PubChem CID: 18815
C18H37Cl

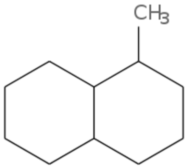

Trans-anti-1-methyl-decahydronaphthalene
PubChem CID: 34193
C11H20

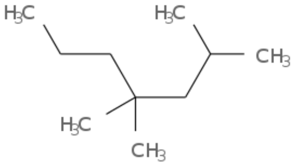

2,2,4-Trimethylheptane
PubChem CID: 77653
C10H22

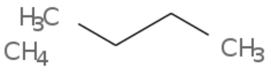

Alkanes
PubChem CID: 94630
C5H14

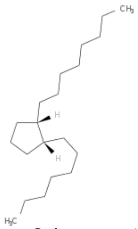

8-Isoprostane
PubChem CID: 107873
C20H40

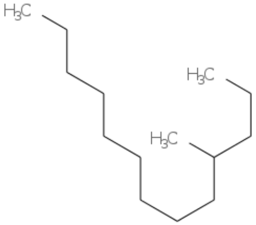

4-Methyltridecane
PubChem CID: 117325
C14H30

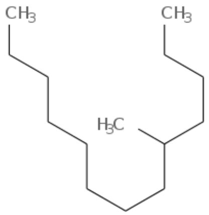

5-Methyltridecane
PubChem CID: 520182
C14H30

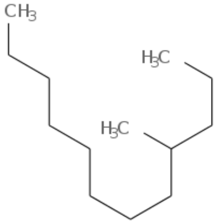

4-Methyldodecane
PubChem CID: 521958
C13H28

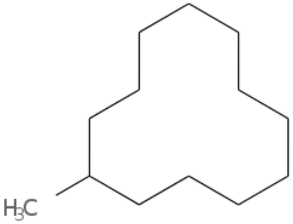

Methylcyclododecane
PubChem CID: 524446
C13H26

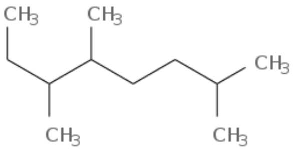

2,5,6-Trimethyloctane
PubChem CID: 545571
C11H24

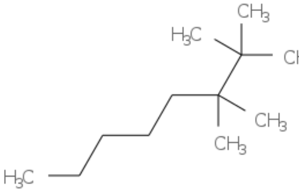

Pentamethylheptane
PubChem CID: 3033849
C12H26

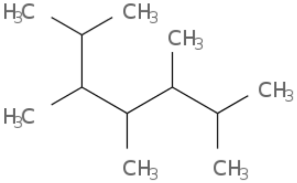

2,3,4,5,6-Pentamethylheptane
PubChem CID: 10910000
C12H26

Cluster 4 (18 VOCs)

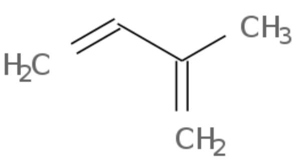

C00046784
Isoprene
PubChem CID: 6557
C5H8

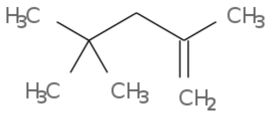

2,4,4-Trimethyl-1-pentene
PubChem CID: 7868
C8H16

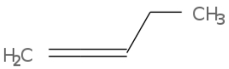

1,2-Pentadiene
PubChem CID: 11588
C5H8

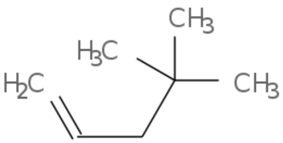

4,4-Dimethyl-1-pentene
PubChem CID: 12984
C7H14

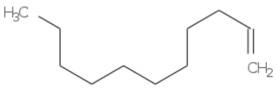

1-Undecene
PubChem CID: 13190
C11H22

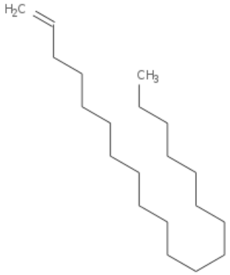

1-Eicosene
PubChem CID: 18936
C20H40

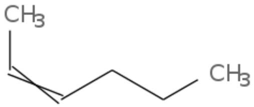

2-Hexene
PubChem CID: 19966
C6H12

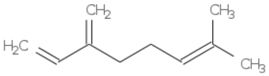

C00000853
Myrcene
PubChem CID: 31253
C10H16

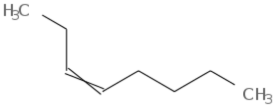

3-Octene
PubChem CID: 32935
C8H16

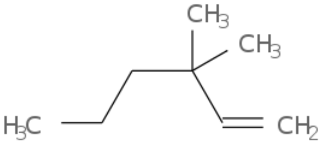

3,3-Dimethylhex-1-ene
PubChem CID: 137924
C8H16

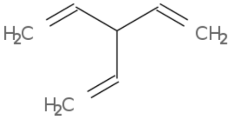

1,4-Pentadiene
PubChem CID: 141289
C7H10

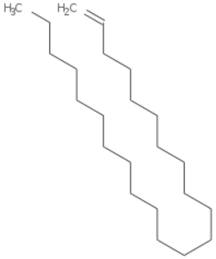

Tricosene
PubChem CID: 181154
C23H46

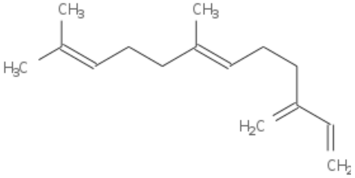

C00003131
beta-Farnesene
PubChem CID: 5281517
C15H24

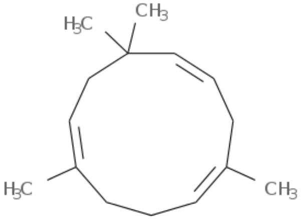

C00003147
alpha-Caryophyllene
PubChem CID: 5281520
C15H24

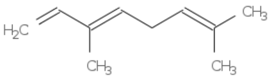

Ocimene
PubChem CID: 5281553
C10H16

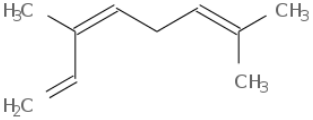

C00029335
beta-Ocimene
PubChem CID: 5320250
C10H16

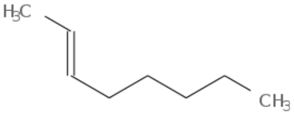

2-Octene
PubChem CID: 5364448
C8H16

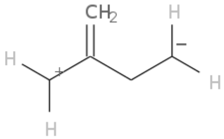

2-Methylbut-1-ene
PubChem CID: 53627566
C5H8

Cluster 5 (21 VOCs)

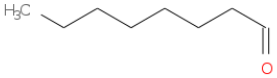

C00030880
Octanal
PubChem CID: 454
C8H16O

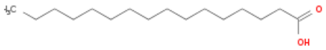

Hexadecanoic acid
PubChem CID: 985
C16H32O2

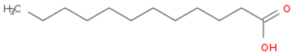

Dodecanoic acid
PubChem CID: 3893
C12H24O2

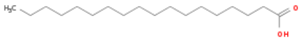

C00001238
Octadecanoic acid
PubChem CID: 5281
C18H36O2

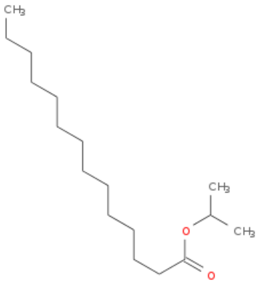

Isopropyl myristate
PubChem CID: 8042
C17H34O2

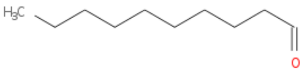

C00030099
Decanal
PubChem CID: 8175
C10H20O

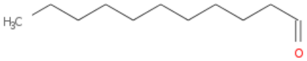

C00032442
Undecanal
PubChem CID: 8186
C11H22O

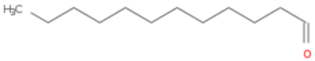

Dodecanal
PubChem CID: 8194
C12H24O

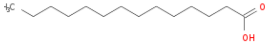

C00001228
Tetradecanoic acid
PubChem CID: 11005
C9H20

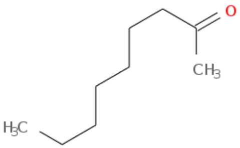

C00029463
2-Nonanone
PubChem CID: 13187
C9H18O

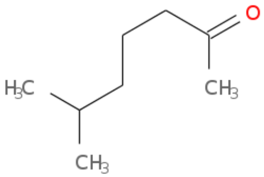

6-Methylheptan-2-one
PubChem CID: 13572
C8H16O

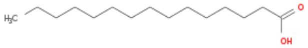

C00007423
Pentadecanoic acid
PubChem CID: 13849
C15H30O2

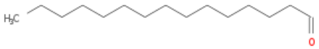

C00030959
Pentadecanal
PubChem CID: 17697
C15H30O

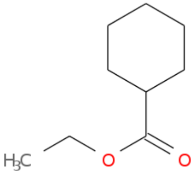

Ethyl cyclohexanecarboxylate
PubChem CID: 18686
C9H16O2

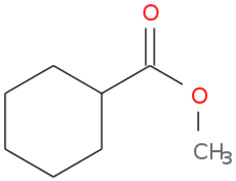

Methyl cyclohexanecarboxylate
PubChem CID: 20748
C8H14O2

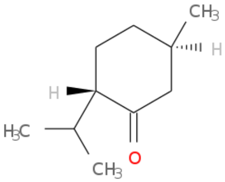

p-Menthone
PubChem CID: 26447
C10H18O

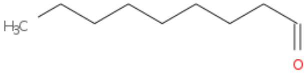

C00030828
Nonanal
PubChem CID: 31289
C9H18O

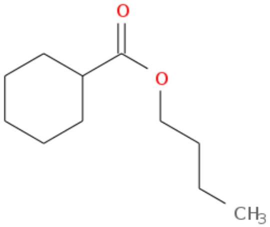

Butyl cyclohexanecarboxylate
PubChem CID: 81040
C11H20O2

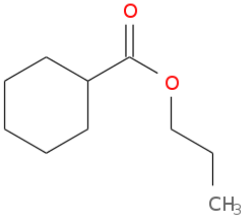

Propyl cyclohexanecarboxylate
PubChem CID: 81222
C10H18O2

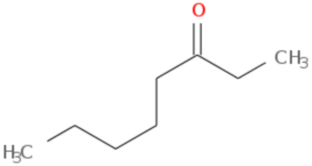

Octan-3-one
PubChem CID: 246728
C8H16O

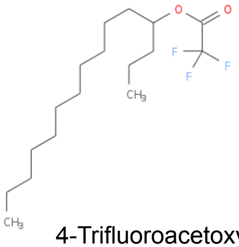

4-Trifluoroacetoxypentadecane
PubChem CID: 543277
C17H31F3O2

Cluster 6 (25 VOCs)

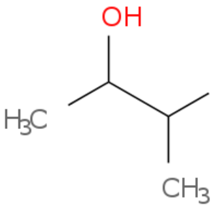

C00050411
2,3-Butanediol
PubChem CID: 262
C4H10O2

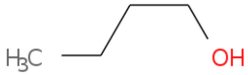

1-Butanol
PubChem CID: 263
C4H10O

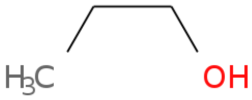

Propanol
PubChem CID: 1031
C3H8O

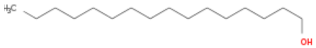

1-Hexadecanol
PubChem CID: 2682
C16H34O

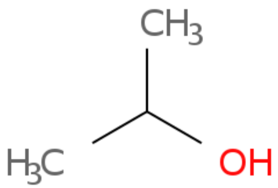

2-Propanol
PubChem CID: 3776
C3H8O

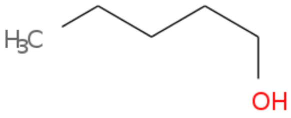

Pentanol
PubChem CID: 6276
C5H12O

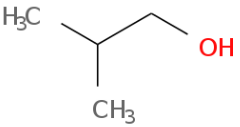

2-Methyl-1-propanol
PubChem CID: 6560
C4H10O

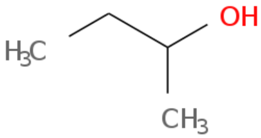

2-Butanol
PubChem CID: 6568
C4H10O

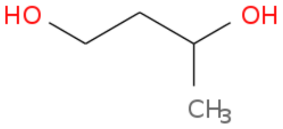

1, 3-butanediol
PubChem CID: 7896
C4H10O2

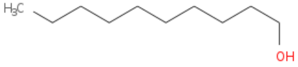

C00030100
Decanol
PubChem CID: 8174
C10H22O

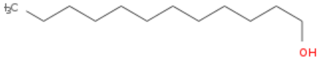

C00030152
1-Dodecanol
PubChem CID: 8193
C12H26O

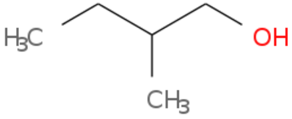

C00050415
2-Methyl-1-butanol
PubChem CID: 8723
C5H12O

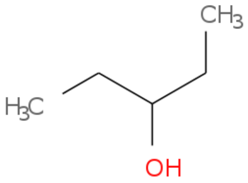

3-Pentanol
PubChem CID: 11428
C5H12O

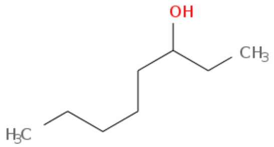

C00035495
3-Octanol
PubChem CID: 11527
C8H18O

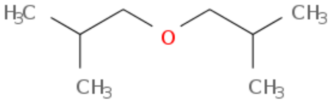

Isobutylether
PubChem CID: 12346
C8H18O

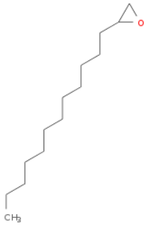

2-Dodecyloxirane
PubChem CID: 18604
C14H28O

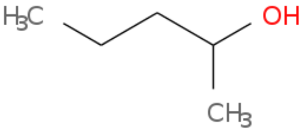

2-Pentanol
PubChem CID: 22386
C5H12O

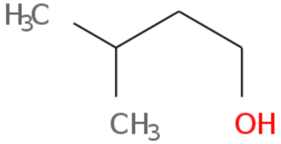

3-Methyl-1-butanol
PubChem CID: 31260
C5H12O

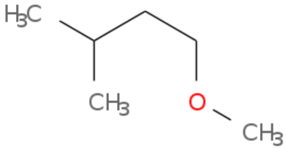

Butane-1-methoxy-3-methyl
PubChem CID: 136445
C6H14O

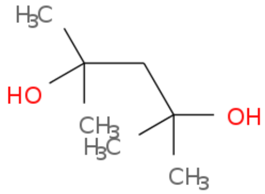

2,4-Dimethylpentane
PubChem CID: 141153
C7H16O2

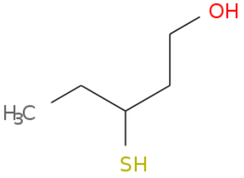

3-Sulfanyl-pentan-1-ol
PubChem CID: 480012
C5H12OS

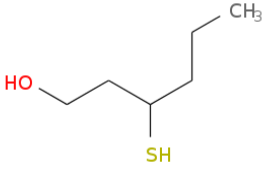

3-Sulfanylhexan-1-ol
PubChem CID: 521348
C6H14OS

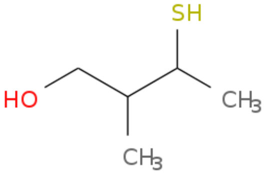

2-Methyl-3-sulfanylbutan-1-ol
PubChem CID: 6430892
C5H12OS

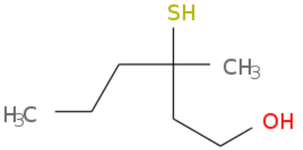

3-Methyl-3-sulfanylhexan-1-ol
PubChem CID: 10130039
C7H16OS

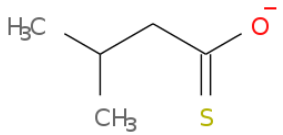

S-Methyl 3-methylbutanethioate
PubChem CID: 21274855
C5H9OS-

Cluster 7 (47 VOCs)

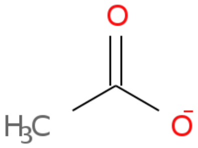

Acetate
PubChem CID: 175
C2H3O2-

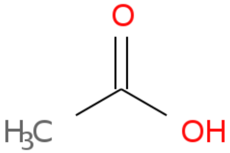

Acetic acid
PubChem CID: 176
C2H4O2

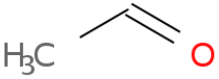

C00007392
Acetaldehyde
PubChem CID: 177
C2H4O

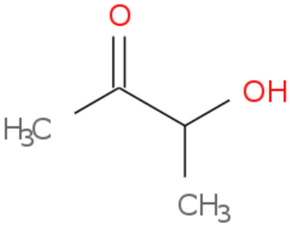

C00050424
Acetoin
PubChem CID: 179
C4H8O2

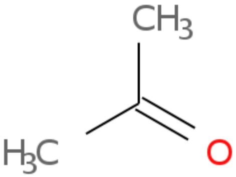

C00048304
Acetone
PubChem CID: 180
C3H6O

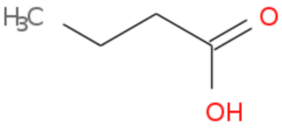

Butanoic acid
PubChem CID: 264
C4H8O2

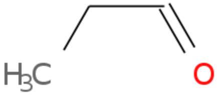

C00050492
Propanal
PubChem CID: 527
C3H6O

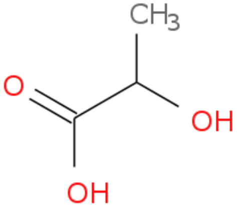

Propanoic acid
PubChem CID: 612
C3H6O3

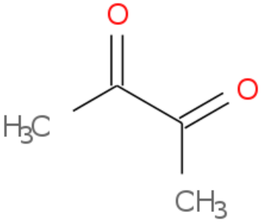

C00050437
2,3-Butanedione
PubChem CID: 650
C4H6O2

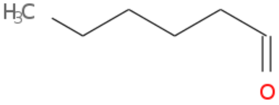

C00000357
Hexanal
PubChem CID: 6184
C6H12O

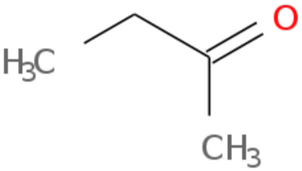

2-Butanone
PubChem CID: 6569
C4H8O

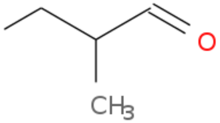

2-Methyl-butanal
PubChem CID: 7284
C5H10O

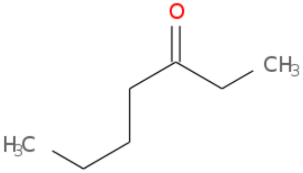

3-Heptanone
PubChem CID: 7802
C7H14O

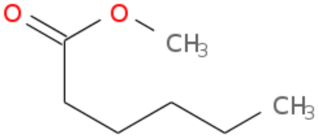

Methyl hexanoate
PubChem CID: 7824
C7H14O2

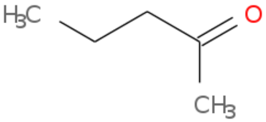

2-Pentanone
PubChem CID: 7895
C5H10O

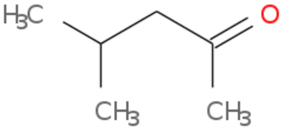

C00051562
Methyl isobutyl ketone
PubChem CID: 7909
C6H12O

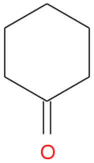

Cyclohexanone
PubChem CID: 7967
C6H10O

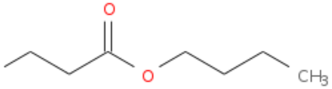

1-Chloro-3-methylbutane
PubChem CID: 7983
C8H16O2

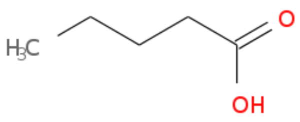

Pentanoic acid
PubChem CID: 7991
C5H10O2

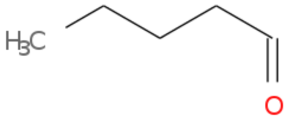

Pentanal
PubChem CID: 8063
C5H10O

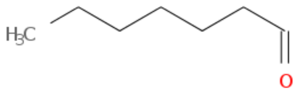

Heptanal
PubChem CID: 8130
C7H14O

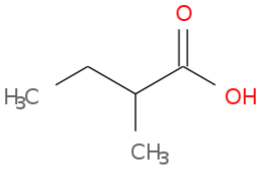

C00029461
2-Methylbutanoic acid
PubChem CID: 8314
C5H10O2

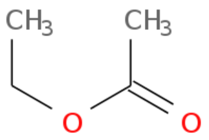

C00001308
Ethyl acetate
PubChem CID: 8857
C4H8O2

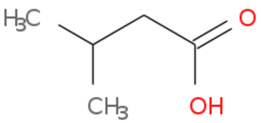

C00001189
3-Methylbutanoic acid
PubChem CID: 10430
C5H10O2

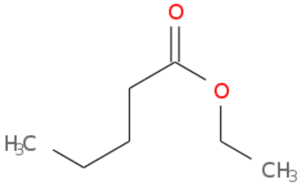

Ethyl valerate
PubChem CID: 10882
C7H14O2

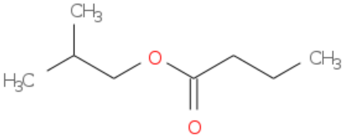

2-Methylpropyl butanoate
PubChem CID: 10885
C8H16O2

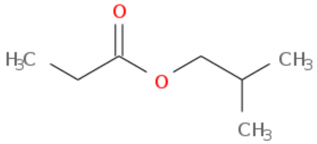

2-Methylpropyl propanoate
PubChem CID: 10895
C7H14O2

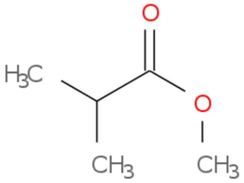

Methyl 2-methylpropanoate
PubChem CID: 11039
C5H10O2

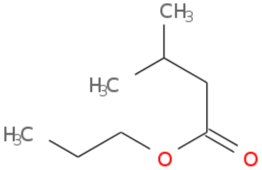

Propyl 3-methylbutanoate
PubChem CID: 11176
C8H16O2

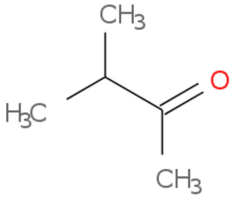

3-Methyl-butan-2-one
PubChem CID: 11251
C5H10O

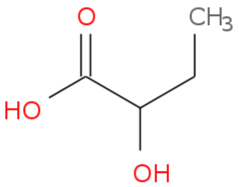

alpha-Hydroxybutyric acid
PubChem CID: 11266
C4H8O3

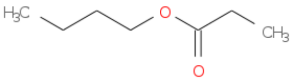

Butyl propanoate
PubChem CID: 11529
C7H14O2

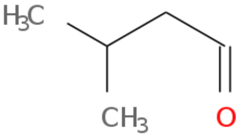

C00048949
3-Methyl-butanal
PubChem CID: 11552
C5H10O

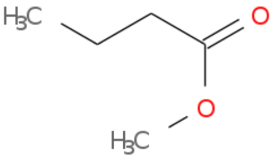

Methyl butanoate
PubChem CID: 12180
C5H10O2

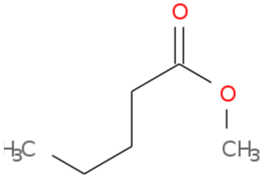

Methyl pentanoate
PubChem CID: 12206
C6H12O2

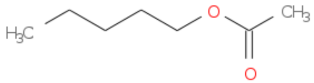

Pentyl acetate
PubChem CID: 12348
C7H14O2

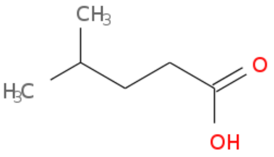

4-Methylpentanoic acid
PubChem CID: 12587
C6H12O2

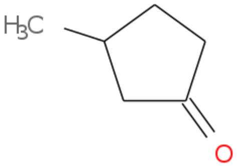

3-Methyl-cyclopentanone
PubChem CID: 15650
C6H10O

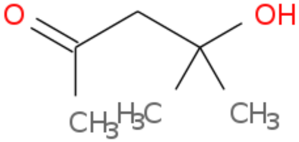

4-Hydroxy-4-methyl-2-pentanone
PubChem CID: 31256
C6H12O2

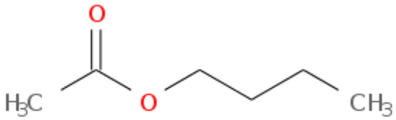

C00048958
Butyl acetate
PubChem CID: 31272
C6H12O2

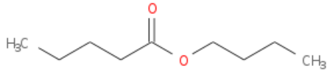

Butyl pentanoate
PubChem CID: 61137
C9H18O2

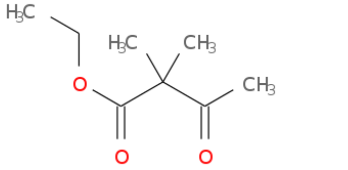

Ethyl 2,2-dimethyl-3-oxobutanoate
PubChem CID: 69002
C8H14O3

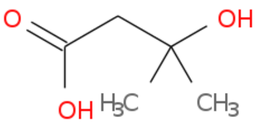

3-Hydroxyisovaleric acid
PubChem CID: 69362
C5H10O3

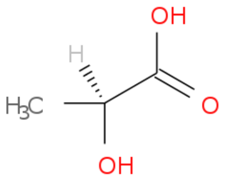

C00050478
Lactic acid
PubChem CID: 107689
C3H6O3

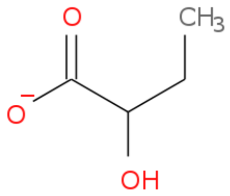

2-Hydroxy-butyrate
PubChem CID: 4071895
C4H7O3-

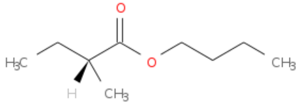

Butyl (2R)-2-methylbutanoate
PubChem CID: 6997354
C9H18O2

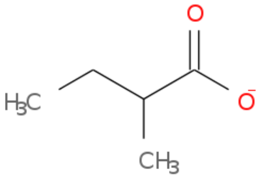

2-Methylbutanoate
PubChem CID: 22253297
C5H9O2-

Cluster 8 (15 VOCs)

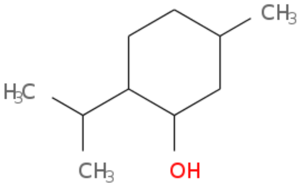

5-Methyl-2-(1-methylethyl)-cyclohexanol
PubChem CID: 1254
C10H20O

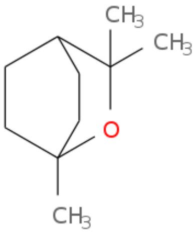

C00000136
1,8-Cineole
PubChem CID: 2758
C10H18O

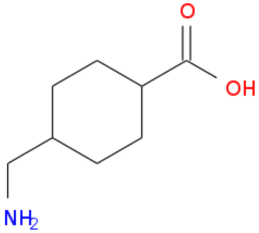

Cyclohexanecarboxylic acid
PubChem CID: 5526
C8H15NO2

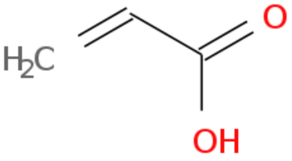

C00034423
2-Propenoic acid
PubChem CID: 6581
C3H4O2

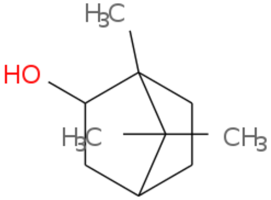

C00003028
Borneol
PubChem CID: 64685
C10H18O2

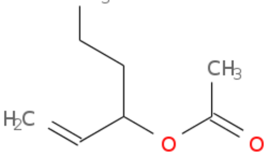

3-Hexenyl acetate
PubChem CID: 118262
C3H6O

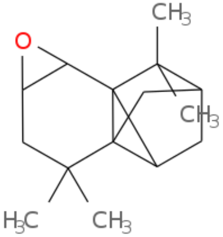

Cyclolongifolene oxide
PubChem CID: 560863
C15H22O

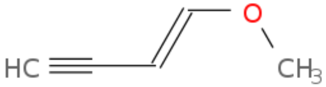

C00030482
Hexyl tiglate
PubChem CID: 637523
C11H20O2

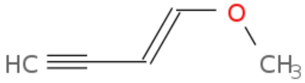

1-Methoxy-1-buten-3-yne
PubChem CID: 643188
C5H6O

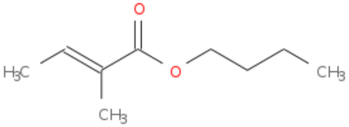

Butyl tiglate
PubChem CID: 5352450
C9H16O2

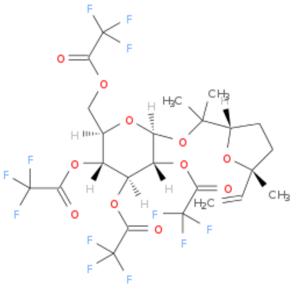

Linalool oxide (furanoid)
PubChem CID: 6428167
C24H24F12O11

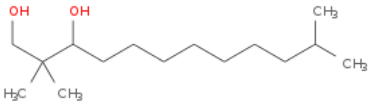

C00011411
2,6,10-Trimethyldodecane
PubChem CID: 44145032
C15H32O2

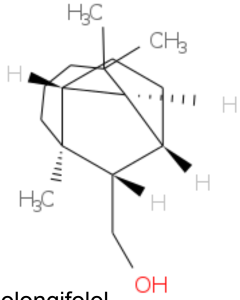

Isolongifolol
PubChem CID: 12311096
C15H26O

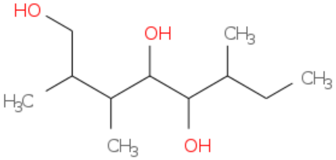

2,3,6-Trimethyloctane
PubChem CID: 58940158
C11H24O3

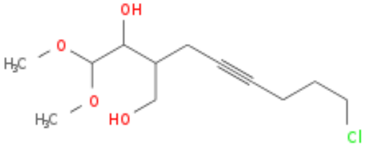

1,4-Dimethoxy-2,3-butanediol
PubChem CID: 71403024
C12H29ClO8

Cluster 9 (42 VOCs)

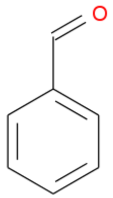

C00034452
Benzaldehyde
PubChem CID: 240
C7H6O

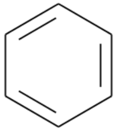

Benzene
PubChem CID: 241
C6H6

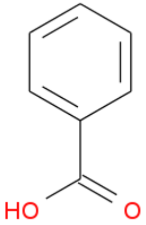

C00000207
Benzoic acid
PubChem CID: 243
C7H6O2

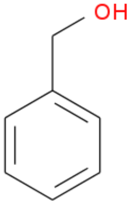

C00029811
Benzyl alcohol
PubChem CID: 244
C7H8O

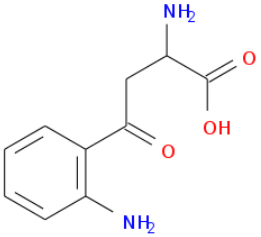

C00051181
Kynurenine
PubChem CID: 846
C10H12N2O3

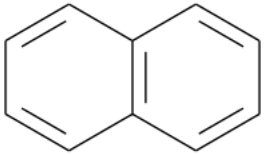

C00001259
Naphthalene
PubChem CID: 931
C10H8

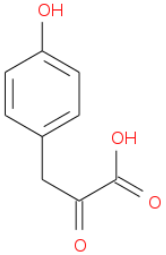

C00007512
p-Hydroxyphenylpyruvic acid
PubChem CID: 979
C9H8O4

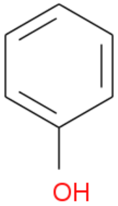

C00002664
Phenol
PubChem CID: 996
C6H6O

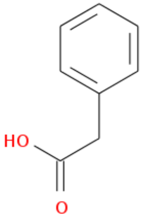

C00000750
Phenylacetic acid
PubChem CID: 999
C8H8O2

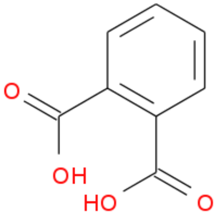

Phthalic acid
PubChem CID: 1017
C8H6O4

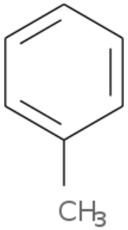

Toluene
PubChem CID: 1140
C7H8

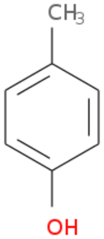

C00002645
4-Methylphenol
PubChem CID: 2879
C7H8O

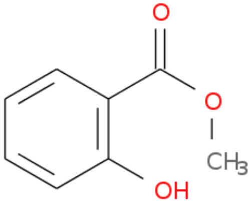

C00030767
Methyl salicylate
PubChem CID: 4133
C8H8O3

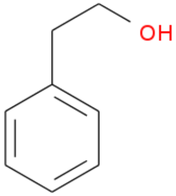

C00002663
2-Phenylethanol
PubChem CID: 6054
C8H10O

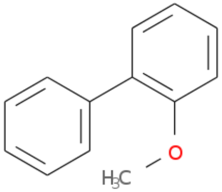

o-Phenylanisole
PubChem CID: 6835
C13H12O

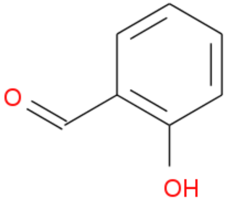

C00031273
Salicylaldehyde
PubChem CID: 6998
C7H6O2

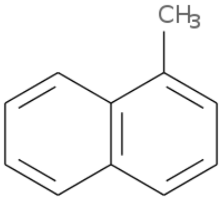

C00050647
1-Methylnaphthalene
PubChem CID: 7002
C11H10

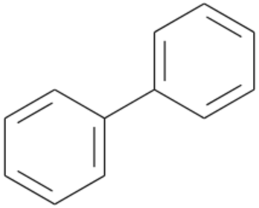

Biphenyl
PubChem CID: 7095
C12H10

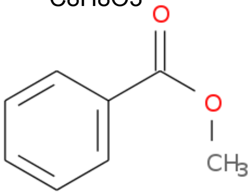

C00034054
Methyl benzoate
PubChem CID: 7150
C8H8O2

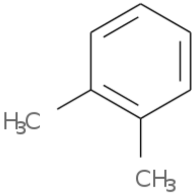

C00035778
1,3-Dimethylbenzene
PubChem CID: 7237
C8H10

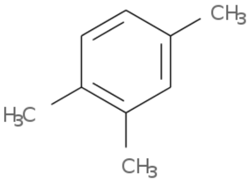

Trimethylbenzene
PubChem CID: 7247
C9H12

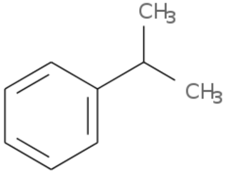

3-(1-methylethyl)-benzene
PubChem CID: 7406
C9H12

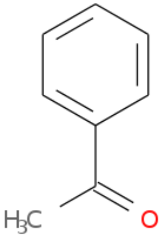

1-Phenyl-ethanone
PubChem CID: 7410
C8H8O

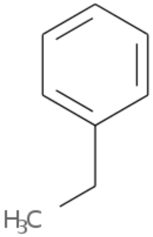

Ethylbenzene
PubChem CID: 7500
C8H10

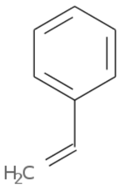

C00037855
Styrene
PubChem CID: 7501
C_8H_8

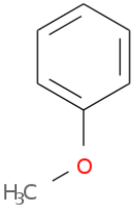

Anisole
PubChem CID: 7519
C_7H_8O

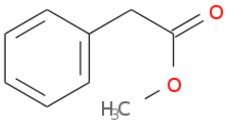

C00051576
Methyl phenylacetate
PubChem CID: 7559
$C_9H_{10}O_2$

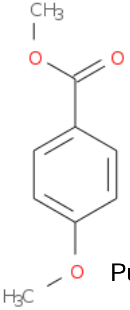

C00030761
Methyl p-anisate
PubChem CID: 8499
$C_9H_{10}O_3$

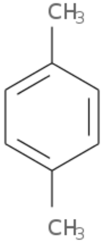

1,4-Xylene
PubChem CID: 7809
C_8H_{10}

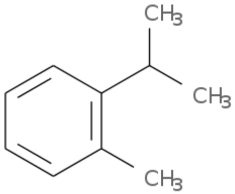

1-Methyl-2-(1-methylethyl)-benzene
PubChem CID: 10703
$C_{10}H_{14}$

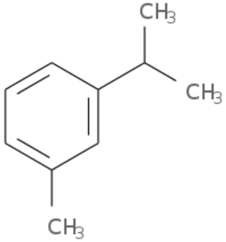

beta-Cymene
PubChem CID: 10812
$C_{10}H_{14}$

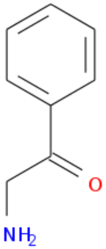

2-Aminoacetophenone
PubChem CID: 11952
C_8H_9NO

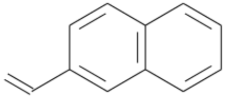

2-Ethenylnaphtalene
PubChem CID: 13230
$C_{12}H_{10}$

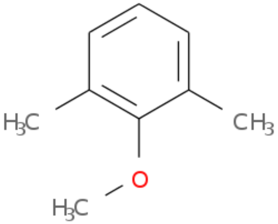

2-Methoxy-1,3-dimethylbenzene
PubChem CID: 66088
$C_{15}H_{24}$

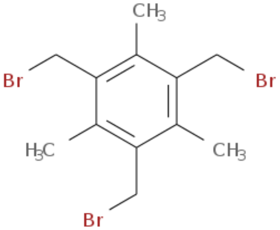

1,2,4-Trimethylbenzene
PubChem CID: 89140
$C_{12}H_{15}Br_3$

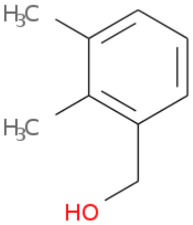

Benzenemethanol
PubChem CID: 96208
$C_9H_{12}O$

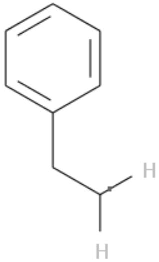

2-Phenylethyl alcohol
PubChem CID: 146323
C_8H_9

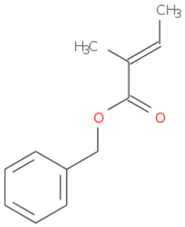

C00048327
Benzyl tiglate
PubChem CID: 250096
$C_{12}H_{14}O_2$

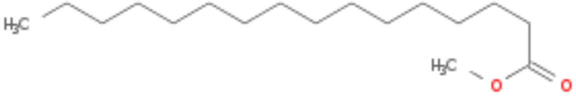

C00030755
Methyl hexadecanoate
PubChem CID: 470382
$C_{17}H_{44}FNO_4$

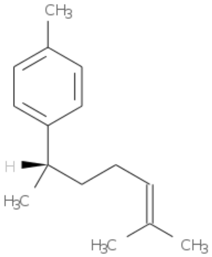

C00046607
alpha-Curcumene
PubChem CID: 3083834
$C_{15}H_{22}$

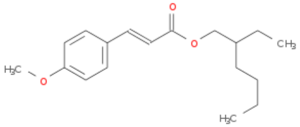

2-Ethylhexyl-4-methoxy-cinnamate
PubChem CID: 5355130
$C_{18}H_{26}O_3$

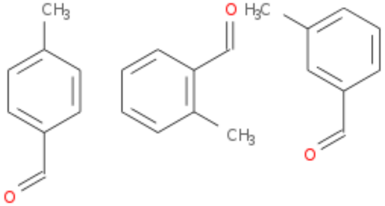

Tolualdehyde
PubChem CID: 24832083
C_8H_8O

Cluster 10 (14 VOCs)

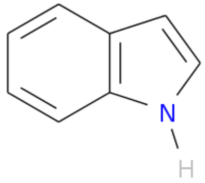

C00001418
Indole
PubChem CID: 798
C8H7N

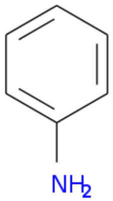

Aniline
PubChem CID: 6115
C6H7N

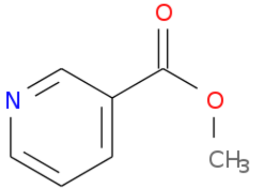

C00044235
Methyl nicotinate
PubChem CID: 7151
C7H7NO2

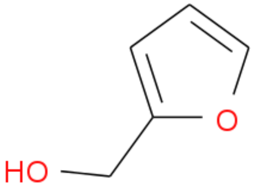

Furanmethanol
PubChem CID: 7361
C5H6O2

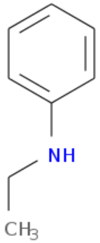

N-Ethylaniline
PubChem CID: 7670
C8H11N

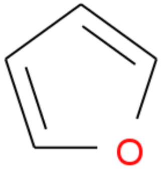

Furan
PubChem CID: 8029
C4H4O

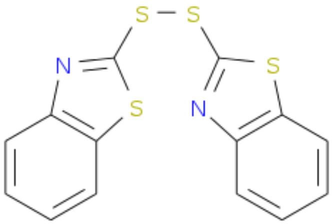

Benzothiazole
PubChem CID: 8447
C14H8N2S4

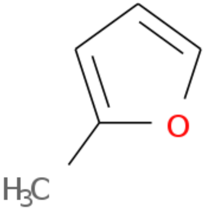

C00048276
2-Methylfuran
PubChem CID: 10797
C5H6O

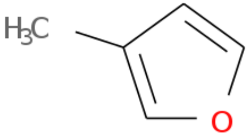

3-Methylfuran
PubChem CID: 13587
C5H6O

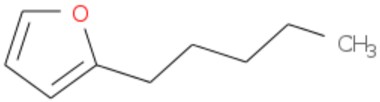

C00034763
2-Pentylfuran
PubChem CID: 19602
C9H14O

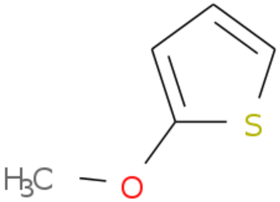

2-Methoxytiophene
PubChem CID: 85610
C5H6OS

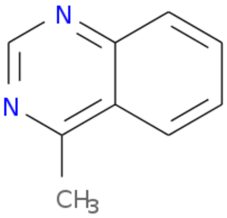

4-Methyl-quinazo- line
PubChem CID: 241520
C9H8N2

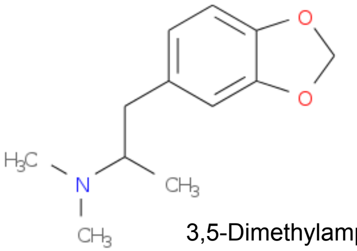

3,5-Dimethylamphetamine
PubChem CID: 551630
C12H17NO2

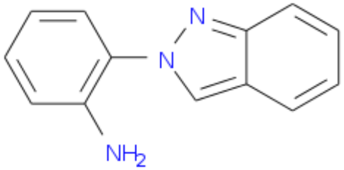

2-Indazol-2-ylphenylamine
PubChem CID: 610670
C13H11N3

Cluster 11 (30 VOCs)

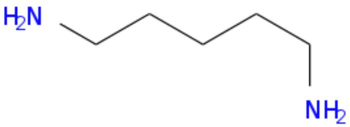

C00001403
Cadaverine
PubChem CID: 273
C5H14N2

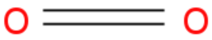

Carbon dioxide
PubChem CID: 280
CO2

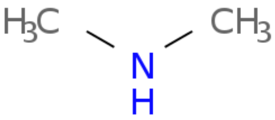

C00050440
Dimethylamine
PubChem CID: 674
C2H7N

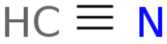

Hydrogen cyanide
PubChem CID: 768
CHN

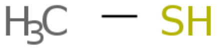

Methylmercaptan
PubChem CID: 878
CH4S

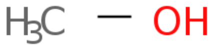

C00050480
Methanol
PubChem CID: 887
CH4O

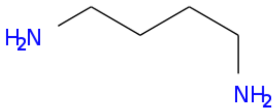

C00001428
Putrescine
PubChem CID: 1045
C4H12N2

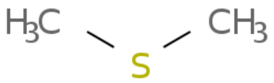

Benzene
PubChem CID: 1068
Dimethyl sulfide
C2H6S

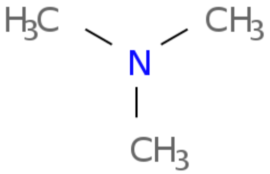

C00001433
Trimethylamine
PubChem CID: 1146
C3H9N

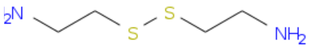

Cystamine
PubChem CID: 2915
C4H12N2S2

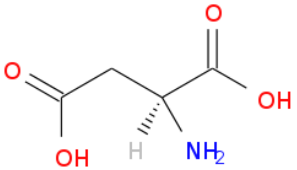

Aspartic acid
PubChem CID: 5960
C4H7NO4

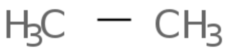

Ethane
PubChem CID: 6324
C2H6

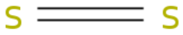

Carbon disulfide
PubChem CID: 6348
CS2

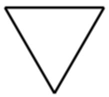

Cyclopropane
PubChem CID: 6351
C3H6

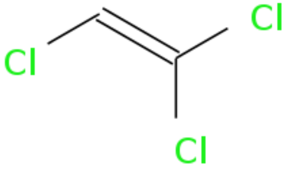

Trichloroethylene
PubChem CID: 6575
C2HCl3

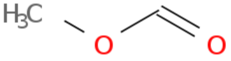

Methyl formate
PubChem CID: 7865
C2H4O2

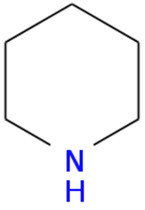

Piperidine
PubChem CID: 8082
C7H8

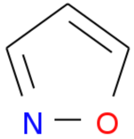

Isoxazole
PubChem CID: 9254
C3H3NO

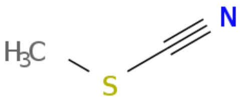

Methyl thiocyanide
PubChem CID: 11168
C2H3NS

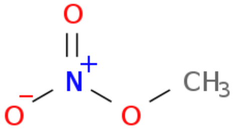

Methyl nitrate
PubChem CID: 11724
CH3NO3

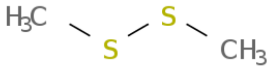

C00001245
Dimethyl disulfide
PubChem CID: 12232
C2H6S2

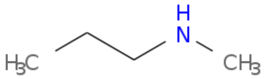

N-Methyl-2-methylpropylamine
PubChem CID: 12315
C4H11N

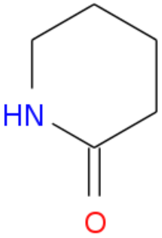

2-Piperidinone
PubChem CID: 12665
C5H9NO

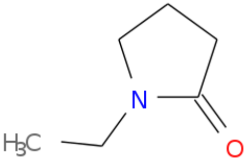

1-Ethyl-2-pyrrolidinone
PubChem CID: 17595
C6H11NO

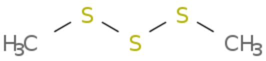

C00001246
Dimethyl trisulfide
PubChem CID: 19310
C2H6S3

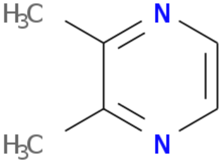

2,3-Dimethyl-pyrazine
PubChem CID: 22201
C6H8N2

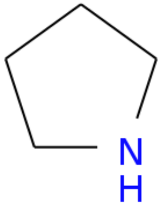

Pyrrolidine
PubChem CID: 31268
C4H9N

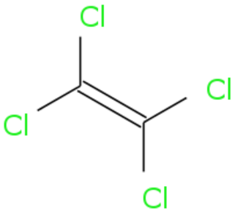

Tetrachloroethyene
PubChem CID: 31373
C2Cl4

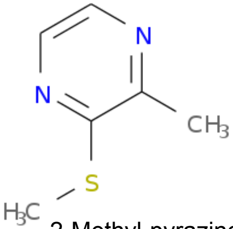

2-Methyl-pyrazine
PubChem CID: 76152
C6H8N2S

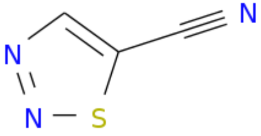

5-Cyano-1,2,3-thiadiazole
PubChem CID: 558078
C3HN3S

Supplementary Figure 3
